# Classical and Bayesian reliability inference for the chen distribution under a block-adaptive progressive hybrid censoring plan

**DOI:** 10.1371/journal.pone.0355463

**Published:** 2026-08-14

**Authors:** Mahmoud M. El-Awady, Hanan Haj Ahmad, Dina A. Ramadan, K. A. Elnagar

**Affiliations:** 1 Basic Sciences Department, Misr Higher Institute for Commerce and Computers, Mansoura, Egypt; 2 Department of Mathematics and Statistics, College of Science, King Faisal University, Hofuf, Al-Ahsa, Saudi Arabia; 3 Department of Mathematics, Faculty of Science, Mansoura University, Mansoura, Egypt; Cairo University, EGYPT

## Abstract

This study investigates statistical inference for lifetime data that are terminated by a block-adaptive progressive hybrid censoring scheme, a design that splits test units across several groups so that experiments finish earlier without losing information. Assuming lifetimes follow the two-parameter Chen distribution, point and interval estimators are obtained for the model parameters, and some key reliability measures are evaluated using both classical and Bayesian frameworks. Bayesian inference is carried out through a Markov chain Monte Carlo procedure that combines Gibbs sampling with Metropolis-Hastings updates for non-standard conditional distributions. The effect of heterogeneity between test blocks on reliability performance is examined. Furthermore, a comprehensive simulation study evaluates the competing estimation methods in terms of bias, mean squared error, average width of the confidence intervals, and coverage probability. The results indicate that the Bayesian estimators achieve the highest accuracy and yield the narrowest interval estimates while maintaining nominal coverage. Two real data applications, from cancer patient survival and electrical breakdown experiments, illustrate the practical advantages of the proposed methodology in reducing test time while preserving essential reliability information.

## 1. Introduction

In lifetime experiments, censoring techniques are widely used to control time and cost limitations, particularly when dealing with highly reliable products that require extended testing durations. In real-world applications, different censoring strategies are designed to optimize efficiency and minimize additional expenditures. Such approaches are commonly utilized in fields such as engineering, including reliability assessments, machine performance analysis, and radio signal processing, as well as biomedical studies. Censoring methods, including Type-I and Type-II censoring, are traditional, where the testing ends at a predetermined time *T* or at a specific number of failures, respectively; see [1]. A combination of these two approaches forms a hybrid censoring scheme.

However, these conventional schemes do not allow for the withdrawal of test units in the middle stages. Therefore, a progressive censoring scheme aimes at addressing the limitations in the flexibility of conventional methods. In progressive type II censoring (PTIIC) schemes, *n* units are placed under observation, with the number of failed units to be recorded set as *m* (m≤n). Let $X_{i}$ (i=1,2,...,m) represent the failure time of the $i^{th}$ unit is recorded for *m* of the *n* units, and let $\left(X_{1},X_{2},...,X_{m}\right)$ denote the PTIIC sample. To draw a PTIIC sample, a predetermined progressive censoring scheme $\left(R_{1},R_{2},...,R_{m}\right)$ is implemented, where after the first failure at time *X*_1:*m*:*n*_, *R*_1_ units are randomly removed from the remaining units. At the time of the second failure, *X*_2:*m*:*n*_, *R*_2_ units are randomly eliminated from the $n-2-R_{1}$ units that still survive, and this process continues similarly until the failure time $m^{th}$ $X_{m:m:n}$. The experiment ends when the remaining units, denoted by $R_{m}=n-m-\sum_{i=1}^{m-1}R_{i}$, are eliminated. Removals $\left(R_{1},R_{2},...,R_{m}\right)$ are predetermined such that condition $m+\sum_{i=1}^{m-1}R_{i}=n$ is satisfied.

A crucial limitation of the PTIIC scheme is that the duration of the experiment *T* is unknown and can be excessively long compared to traditional Type-II censoring schemes, as noted in [2]. As a consequence, the Adaptive Type-II Progressive Censoring scheme (APCS) was proposed, allowing the experimenters to adjust the planned removal schedule of the surviving units during the test.

In the APCS, a predetermined experimental duration *T* > 0 is specified, although the actual test may extend beyond this limit if required. The target number of failures *m* is also predetermined. Although a censoring plan $\left(R_{1},R_{2},...,R_{m}\right)$ is initially set, it is subject to adjustment during the testing process. The APCS provides a balance between reducing the total testing time and collecting sufficient data, including extreme observations, to ensure reliable statistical inference. The termination strategy is based on two possible cases:

**Case (i):** If $T\geq X_{m:m:n}$, then all failures *m* occur before the cutoff time, and the experiment ends at $X_{m:m:n}$, following the original censoring schedule $\left(R_{1},R_{2},...,R_{m}\right)$.
**Case (ii):** If $T<X_{m:m:n}$, the test continues beyond the planned duration. Assume that exactly *J* failures have been observed such that $X_{J:m:n}<T<X_{J+1:m:n}$, where 0≤J≤m−1 and *X*_0:*m*:*n*_ = 0. In this case, the experiment ends with the appearance of the *m*^th^ failure, and the censoring scheme is updated to $\left(R_{1},R_{2},...,R_{J},0,...,0,R_{m}^{*}\right)$, where $R_{m}^{*}=n-m-\sum_{i=1}^{m-1}R_{i}.$

Recently, the APCS has gained considerable attention for its flexibility in balancing time limitations and data completeness in life-testing experiments. Numerous studies have explored its utility across diverse statistical distributions and censoring models. For instance, Elshahhat et al. [3] applied APCS to the Gompertz-Makeham model, while Dutta et al. [4] investigated Bayesian survival analysis for the logistic-exponential distribution under this scheme. The work of Irfan et al. [5] explored optimal planning under APCS for the Kumaraswamy-G family. Further contributions include Bayesian and frequentist inference under improved APCS by Dutta et al. [6], as well as Dutta and Kayal [7], who analyzed dependent competing risks using the adaptive hybrid approach. Gangopadhyay et al. [8] studied inference for the inverse Xgamma distribution under a generalized adaptive progressive scheme, and Nassar et al. [9] conducted a comprehensive analysis for the Weibull distribution within APCS. Bayesian estimation under APCS in the presence of competing risks has been addressed by Ren and Gui [10] for Weibull models and by Ahmad et al. [11] in the context of system performance. Moreover, Almetwally et al. [12] utilized the Maximum Product of Spacings Estimation approach for inference under adaptive censoring frameworks. Additionally, Ahmed and El-Awady [13] investigated estimation techniques for partially accelerated life tests under the APCS setup.

In certain cases, it is not practical to check every unit in a study simultaneously. This challenge may arise due to constraints such as the limited availability of necessary equipment or logistical difficulties in conducting life tests on all units at once. To address this, Ahmadi et al. [14] proposed a new adaptable testing method, named the block censoring scheme. This scheme improves the efficiency of the tests by considerably reducing the total time for tests in life-test experiments. The methodology involves dividing *n* identical test units into *k* groups, where each group consists of $n_{i}(i=1,2,...,k)$ units. Each of these *k* groups is tested in a separate facility under Type-II censoring conditions. The block censoring scheme offers a more practical approach, enabling units to be assessed in multiple groups efficiently. A comprehensive review of this scheme can be found in Zhu [15].

This methodology organizes lifetime tests across distinct groups while operating with Type-II censoring conditions. Modern manufacturing advancements have resulted in products with longer lifespans and improved reliability, making it difficult to obtain sufficient failure data within Type-II censoring. To overcome this limitation, Kumari et al. [16] and Kumari et al. [17] introduced an advanced approach called the block progressive Type-II censoring scheme, which provides increased flexibility and efficiency to other censoring schemes. This method involves testing different groups using multiple facilities, which reduces the overall duration of the experiments. Although testing units are typically assumed to be identical, in practice, variations in failure data between units can occur due to factors such as differences in operating conditions, personnel expertise, environmental influences, and precision of data collection. Consequently, treating these data as non-identical records better reflects real-world variations, highlighting the substantial implications of differences between different facilities (DDF).

Additional developments under this scheme have been explored in recent studies. For instance, Kumari et al. [18] investigated inverted exponentiated distributions, while Kumari et al. [19] focused on pivotal-based inference for the Burr-XII model. Singh et al. [20] examined the unit inverse Weibull distribution, and Kumari et al. [17] provided reliability estimation procedures for the Kumaraswamy distribution under the block progressive Type-II censoring scheme.

Singh et al. [21] developed point and interval estimators for model parameters and reliability metrics within the framework of the block adaptive Type-II hybrid progressive censoring scheme (BAPCS) using Weibull lifetimes.

However, research on BAPCS remains limited. Therefore, this work is motivated by the need to cover the gaps via the development and analysis of more practical and flexible censoring schemes. We focus on the BAPCS, which merges the strengths of block censoring, hybrid censoring, and the progressive withdrawal framework. The novelty of this work lies in the integration of the BAPCS with the Chen distribution, which is known for its ability to model non-monotonic hazard rate trends. To our knowledge, no previous study has investigated inference analysis for the Chen distribution under the BAPCS, nor has it addressed the impact of DDF in such a context. By employing both maximum likelihood estimation (MLE), maximum product of spacings estimation (MPSE), and Bayesian inference, we aim to provide robust point and interval estimators for the model parameters and reliability indices.

**Fig 1:**
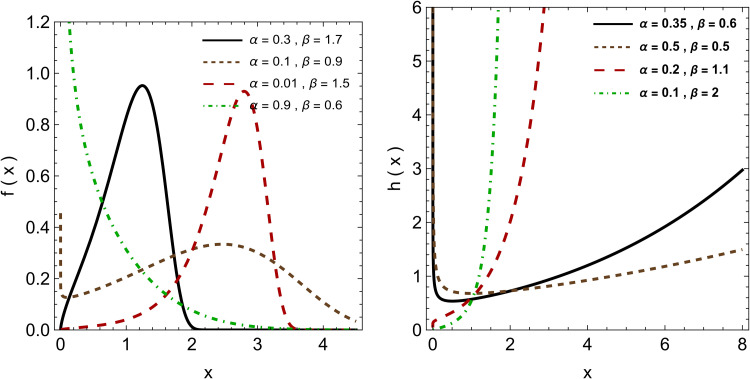
Probability density function and hazard function of the Chen distribution under various parameter values.

The Chen distribution, first introduced by Chen [22], is a flexible and versatile model widely used in the analysis of lifetime and reliability data. A key advantage of this distribution is its ability to capture diverse shapes for both the probability density and hazard functions. The pdf can be decreasing, unimodal, or decreasing-increasing-decreasing, while the hazard function can be either increasing or bathtub-shaped, as shown in Figure 1. This flexibility makes the Chen distribution particularly suitable for modeling failure times of highly reliable products, mechanical systems, and biological processes.

In recent years, the Chen distribution has gained significant attention due to its mathematical tractability and its adaptability to different censoring and truncation schemes. Its parameters offer intuitive control over the scale and shape of the distribution, allowing it to capture complex aging behaviors and heterogeneity in the data. These features make it a compelling choice for applications in industrial reliability testing, biomedical survival analysis, and engineering systems.

Let *X* be a random variable following the Chen distribution. The probability density function (PDF) and the cumulative distribution function (CDF) of *X* are defined as follows:


$$\begin{array}{c} f(x;\beta ,\alpha )=\alpha \beta x^{\beta -1}e^{x^{\beta }}e^{\alpha (1-e^{x^{\beta }})}, \end{array}$$ (1)

$$\begin{array}{c} F(x;\beta ,\alpha )=1-e^{\alpha (1-e^{x^{\beta }})}, \end{array}$$ (2)

respectively. where β>0 is the shape parameter and α>0 is the scale parameter of the model.

For more information and statistical properties of the Chen distribution, refer to Ahmed et al. [23]. Reliability indices are vital in survival analysis, such as the survival function (SF) and the hazard rate function (HRF) which are given by:


$$\begin{array}{c} S(x;\beta ,\alpha )=e^{\alpha (1-e^{x^{\beta }})}, \end{array}$$

and


$$\begin{array}{c} h(x;\beta ,\alpha )=\alpha \beta x^{\beta -1}e^{x^{\beta }}. \end{array}$$

In the context of this study, the Chen distribution is adopted as the baseline model for developing efficient estimation procedures under the BAPCS. Its suitability for modeling a wide range of lifetime behaviors aligns well with the objectives of this paper, which focuses on improving the accuracy and practicality of statistical inference in censored reliability experiments.

The motivation for this work arises from several practical and methodological needs in modern reliability testing. In real-life applications, lifetime experiments frequently involve multiple testing facilities, heterogeneous operating conditions, and strict time constraints. Conventional censoring schemes often fail to address these challenges. Therefore, the present study is motivated by the following key points:

1. Traditional Type-I, Type-II, and hybrid censoring designs do not allow adaptive withdrawal of units or multi-facility coordination, which limits their efficiency in long-duration life tests. The BAPCS provides a more realistic and time-saving alternative by combining block testing, hybrid termination, and progressive adaptive withdrawal.
2. When identical units are tested across different facilities, variations naturally arise due to environmental, operational, or measurement differences. Ignoring these variations leads to biased and unreliable inference. Modeling group-specific scale parameters $\left(\alpha_{1},\alpha_{2},...,\alpha_{k}\right)$ under BAPCS addresses this important practical issue.
3. Many engineering and biomedical systems exhibit non-monotonic hazard rates (e.g., bathtub-shaped), which cannot be captured adequately by classical models. The Chen distribution is highly flexible and capable of representing multiple hazard-rate patterns, making it well-suited for the BAPCS setting.
4. To the best of our knowledge, no published work has developed classical, MPSE, and Bayesian inference for the Chen distribution under BAPCS while accounting for differential facility effects. This creates a significant methodological gap that the present work aims to fill.
5. Due to the highly nonlinear likelihood under BAPCS, different estimation paradigms (MLE, MPSE, Bayesian) are required to ensure robustness, accuracy, and applicability in both small and moderate sample sizes.

Overall, these motivations underline the importance of proposing a comprehensive inferential framework for the Chen distribution under BAPCS, enabling more accurate and practical reliability assessment in real-world multi-facility testing environments.

The rest of this paper is prepared as follows: Section 2 describes the BAPCS model. Section 3 analyzes the maximum likelihood point and interval estimators, while Section 4 presents Bayesian inference. Section 5 studies the maximum product of spacings estimation method, while Section 6 develops a simulation study to assess the performance of these estimation methods. Real data applications are performed in Section 7, where two real data sets are considered, one from the medical field and the other from electrical engineering. Finally, concluding remarks are presented in Section 8.

## 2. Model description

Several studies have focused on the evolution of censoring schemes to enhance efficiency in reliability experiments. The block censoring scheme proposed by Ahmadi et al. [14] provided a practical means of testing multiple groups under Type-II conditions, later reviewed in detail by Zhu [15]. To address the limitations of conventional Type-II censoring in modern reliability testing, Kumari et al. [16] and [17] introduced the block progressive Type-II censoring scheme, which offers greater flexibility and reduced test duration. Recent works, such as Kumari et al. [18,19], and Singh et al. [20], further extended this framework to different statistical distributions and pivotal inference procedures. Building on these advancements, the present study incorporates the Chen distribution within the block progressive Type-II censoring context to provide a more adaptable model for multi-component reliability systems.

Consider a scenario in which *k* groups and *n* similar units are used with the BAPCS framework. A fixed total experiment time *T* is assumed, hence, according to the APCS, each group, indicated as having a size $n_{i}$ (where *i* = 1, 2,..., *k*), is tested individually, and the sum of the group sizes satisfies $\sum_{i=1}^{k}n_{i}=n$. For the $i^{th}$ group, assume that the actual number of failures obtained is $m_{i}$, and the duration of the experiment for this group is $T_{i}$. In this group, the predefined censoring scheme is represented by the sequence $R_{i}=(R_{i1},R_{i2},...,R_{im_{i}})$. Consider that the failure time of the $i^{th}$ unit is denoted as $X_{im_{i}}$. In the BAPCS method, if $X_{im_{i}}<T_{i}$, the experiment ends at $X_{im_{i}}$, and the censoring scheme is defined as $\left(R_{i1},R_{i2},...,R_{im_{i}}=n_{i}-m_{i}-\sum_{j=1}^{m_{i}-1}R_{ij}\right)$ for the $i^{th}$ testing group. On the other hand, if $X_{im_{i}}>T_{i}$, there exists a value $J_{i}$ such that $X_{iJ_{i}}<T_{i}<X_{iJ_{i}+1}$. In this case, the experiment continues until$X_{im_{i}}$, without unit removal, and the censoring scheme is set with $R_{il}=0$, where $l=J_{i}+1,...,m_{i}-1$ and $R_{im_{i}}=n_{i}-m_{i}-\sum_{j=1}^{J_{i}}R_{ij}$. In the BAPCS approach, the censoring scheme $\left(R_{i1},R_{i2},...,R_{im_{i}}\right)$ is modified to $\left(R_{i1},R_{i2},...,R_{iJ_{i}},0,...,0,R_{im_{i}}^{*}=n_{i}-m_{i}-\sum_{j=1}^{J_{i}}R_{ij}\right)$ for each $i^{th}$ group.

Under the APCS approach, the failure times in the $i^{th}$ group are observed as $X_{i}=(X_{i1},X_{i2},...,X_{im_{i}})$. It is assumed that the lifetimes of the products follow a Chen distribution, with β and $\alpha_{i}$ as the distribution parameters. Taking DDF into account, the lifetimes of the units in the $i^{th}$ group, tested at the $i^{th}$ testing facility, are modeled using a Chen distribution with parameters β and $\alpha_{i}$. The PDF and the CDF are given as:


$$\begin{array}{c} f(x;\beta ,\alpha_{i})=\alpha_{i}\beta x^{\beta -1}e^{x^{\beta }}e^{\alpha_{i}(1-e^{x^{\beta }})}, \end{array}$$ (3)

$$\begin{array}{c} F(x;\beta ,\alpha_{i})=1-e^{\alpha_{i}(1-e^{x^{\beta }})}. \end{array}$$ (4)

It is important to note that various facilities may share similar fundamental operational processes. Therefore, we apply a common parameter β across these facilities. However, distinct parameters $\alpha_{1},\alpha_{2},...,\alpha_{k}$ are introduced to account for the facility-specific effects within the *k* testing groups. These parameters capture the variations observed when testing identical units in different facilities. In essence, the differences in these parameters contribute to the DDF, and accurately estimating these values is crucial for the proper estimation of the DDF function. Furthermore, given that the DDF cannot be disregarded, the parameter $\alpha_{i}$ in Equation (3) can be estimated by using a weighted approach as follows:


$$\begin{array}{c} \hat{\alpha }=\frac{\sum_{i=1}^{k}\omega_{i}\alpha_{i}}{\sum_{i=1}^{k}\omega_{i}} \end{array}$$ (5)

In this context, $\omega_{i}$ (where *i* = 1, 2, ..., *k*) represents the weight coefficient, with $\omega_{i}=\frac{1}{var(\alpha_{i})}$, and $var(\alpha_{i})$ referring to the variance of $\alpha_{i}$.

**Fig 2:**
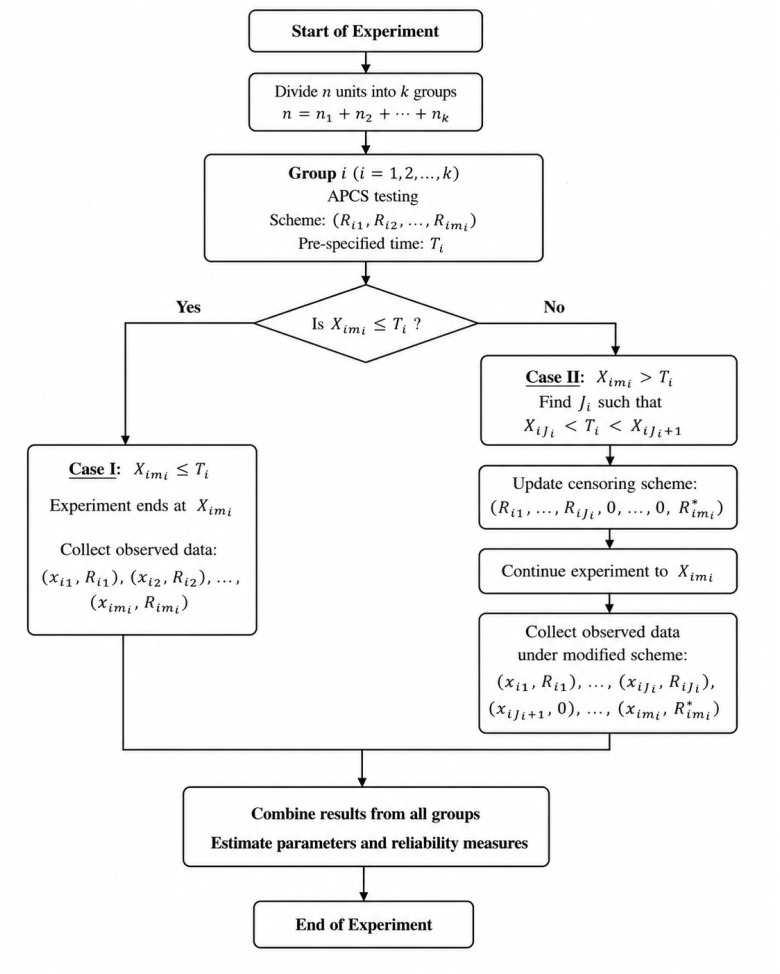
Flowchart of BAPCS for *k* groups, illustrating the experimental procedure and data collection.

The failure times are recorded under the BAPCS, as outlined below in Table 1 and illustrated in Fig 2.

**Table 1:** Failure times with BAPCS outline.

Group	Failure Times	Detailed samples
1	$\vartheta_{1}^{\prime }$	Case−I: ($x_{11},R_{11}),(x_{12},R_{12}),...,(x_{1m_{1}},R_{1m_{1}}),$ i f $X_{1m_{1}}<T_{1}$,
		Case−II: ($x_{11},R_{11}),(x_{12},R_{12}),...,(x_{1J_{1}},R_{1J_{1}}),(x_{1J_{1}+1},0),$
		$\ldots ,(x_{1m_{1}-1},0),(x_{1m_{1}},R_{1m_{1}}^{*})$ i f $X_{1m_{1}}>T_{1}$.
2	$\vartheta_{2}^{\prime }$	Case−I: ($x_{21},R_{22}),(x_{22},R_{22}),...,(x_{2m_{2}},R_{2m_{2}}),$ i f $X_{2m_{2}}<T_{2}$,
		Case−II: ($x_{21},R_{21}),(x_{22},R_{22}),...,(x_{2J_{2}},R_{2J_{2}}),(x_{2J_{2}+1},0),$
		$\ldots ,(x_{2m_{2}-1},0),(x_{2m_{2}},R_{2m_{2}}^{*})$ i f $X_{2m_{2}}>T_{2}$.
⋮	⋮	⋮
*k*	$\vartheta_{k}^{\prime }$	Case−I: ($x_{k1},R_{k2}),(x_{k2},R_{k2}),...,(x_{km_{k}},R_{km_{k}}),$ i f $X_{km_{k}}<T_{k}$,
		Case−II: ($x_{k1},R_{k1}),(x_{k2},R_{k2}),...,(x_{kJ_{k}},R_{kJ_{k}}),(x_{kJ_{k}+1},0),$
		$\ldots ,(x_{km_{k}-1},0),(x_{km_{k}},R_{km_{k}}^{*})$ i f $X_{km_{k}}>T_{k}$.

Table 2 presents a numerical illustration of the terms defined in Table 1. For Group 1 (Case I), the test ends when the last failure time $X_{1m_{1}}=110<T_{1}=120$. For Group 2 (Case II), the experiment continues beyond *T*_2_ = 80 up to $X_{2m_{2}}=120$, with *J*_2_ = 4 determining the transition point. The final removal $R_{im_{i}}$ or $R_{im_{i}}^{*}$ is computed according to the respective BAPCS rule.

**Table 2:** Illustrative example of failure times and removals under BAPCS.

Group	Case Type	Failure Times $\left(x_{ij}\right)$	Removals $\left(R_{ij}\right)$	Notes
1	Case-I ($X_{1m_{1}}<T_{1}$)	18, 35, 60, 90, 110	1, 0, 1, 0, 1	$n_{1}=8,\;T_{1}=120,\;m_{1}=5$
2	Case-II ($X_{2m_{2}}>T_{2}$)	12, 30, 55, 78, 95, 120	2, 1, 0, 0, 0, 1	$n_{2}=10,\;T_{2}=80,\;m_{2}=6,\;J_{2}=4$

## 3. Maximum likelihood estimation

Given the BAPCS illustrated above, assume the failure times $\vartheta_{i}^{\prime }$, where *i* = 1, 2, ..., *k*, represent APCS samples following Chen distribution with parameters β and $\alpha_{i}$ for the test group $i^{th}$. According to the censoring model outlined in Ng et al. [2], the likelihood function for the $i^{th}$ facility is:


$$\begin{array}{cc} L_{i}(\beta ,\alpha_{i},\vartheta_{i}^{\prime }) & =C_{i}\prod_{j=1}^{m_{i}}f(x_{ij};\alpha_{i},\beta )\prod_{j=1}^{J_{i}}{\left[1-F(x_{ij};\alpha_{i},\beta )\right]}^{R_{ij}}\times [1-F(x_{im_{i}};\alpha_{i},\beta ){]}^{R_{im_{i}}^{*}}\\ & =C_{i}\alpha_{i}^{m_{i}}\beta^{m_{i}}\prod_{j=1}^{m_{i}}x_{ij}^{\beta -1}e^{\sum_{j=1}^{m_{i}}x_{ij}^{\beta }}e^{\alpha_{i}\psi_{i}(\beta )}, \end{array}$$

where $C_{i}=(\prod_{j=1}^{m_{i}}n_{i}-j+1-\sum_{l=1}^{\min(j-1,J_{i})}R_{il}$) is the normalizing constant and $\psi_{i}(\beta )=\sum_{j=1}^{m_{i}}(1-e^{x_{ij}^{\beta }})+\sum_{j=1}^{J_{i}}R_{ij}(1-e^{x_{ij}^{\beta }})+R_{im_{i}}^{*}(1-e^{x_{im_{i}}^{\beta }})$.

Consider $\vartheta^{\prime }=(\vartheta_{1}^{\prime },\vartheta_{2}^{\prime },...,\vartheta_{k}^{\prime })$ and $\alpha =(\alpha_{1},\alpha_{2},...,\alpha_{k})$. The likelihood function for the entire BAPCS setup is then expressed as


$$\begin{array}{c} L(\beta ,\alpha ,\vartheta^{\prime })\propto \prod_{i=1}^{k}L_{i}(\beta ,\alpha_{i},\vartheta_{i}^{\prime })=\beta^{\sum_{i=1}^{k}m_{i}}\times \prod_{i=1}^{k}\alpha_{i}^{m_{i}}\times \prod_{i=1}^{k}\prod_{j=1}^{m_{i}}x_{ij}^{\beta -1}\times e^{\sum_{i=1}^{k}\sum_{j=1}^{m_{i}}x_{ij}^{\beta }}e^{\sum_{i=1}^{k}\alpha_{i}\psi_{i}(\beta )}. \end{array}$$ (6)

Hence, the log-likelihood function is as follows:


$$\begin{array}{c} \ell (\beta ,\alpha ,\vartheta^{\prime })\propto \ln(\beta )\sum_{i=1}^{k}m_{i}+\sum_{i=1}^{k}m_{i}\ln(\alpha_{i})+(\beta -1)\sum_{i=1}^{k}\sum_{j=1}^{m_{i}}\ln(x_{ij})+\sum_{i=1}^{k}\sum_{j=1}^{m_{i}}x_{ij}^{\beta }+\sum_{i=1}^{k}\alpha_{i}\psi_{i}(\beta ). \end{array}$$ (7)

Consequently, the MLEs for β and $\alpha_{i}$, *i* = 1, 2, ..., *k*, denoted as $\hat{\beta }$ and $\hat{\alpha }=(\hat{\alpha_{1}},\hat{\alpha_{2}},...,\hat{\alpha_{k}})$, respectively, are found as solutions to the following equations:


$$\begin{array}{c} \frac{\partial \ell }{\partial \beta }=\frac{\sum_{i=1}^{k}m_{i}}{\beta }+\sum_{i=1}^{k}\sum_{j=1}^{m_{i}}\ln(x_{ij})+\sum_{i=1}^{k}\sum_{j=1}^{m_{i}}x_{ij}^{\beta }\ln(x_{ij})+\sum_{i=1}^{k}\alpha_{i}\psi_{i}^{\prime }(\beta )=0, \end{array}$$ (8)

$$\begin{array}{c} \frac{\partial \ell }{\partial \alpha_{i}}=\frac{\sum_{i=1}^{k}m_{i}}{\alpha_{i}}+\sum_{i=1}^{k}\psi_{i}(\beta )=0, \end{array}$$ (9)

where $\psi_{i}^{\prime }(\beta )=-\sum_{j=1}^{m_{i}}x_{ij}^{\beta }\ln(x_{ij})e^{x_{ij}^{\beta }}-\sum_{j=1}^{J_{i}}R_{ij}x_{ij}^{\beta }\ln(x_{ij})e^{x_{ij}^{\beta }}-R_{im_{i}}^{*}x_{im_{i}}^{\beta }\ln(x_{im_{i}})e^{x_{im_{i}}^{\beta }}.$

The MLEs for $\alpha_{i}$ can be obtained directly from Equation (9), and is given by


$$\begin{array}{c} {\hat{\alpha }}_{i}=-\frac{m_{i}}{\psi_{i}(\beta )},\;i=1,...,k. \end{array}$$ (10)

Substituting Equation (10) into Equation (8) reduces the system of likelihood equations to a univariate nonlinear equation in the parameter β.


$$\begin{array}{c} \frac{\partial \ell }{\partial \beta }=\frac{\sum_{i=1}^{k}m_{i}}{\beta }+\sum_{i=1}^{k}\sum_{j=1}^{m_{i}}\ln(x_{ij})+\sum_{i=1}^{k}\sum_{j=1}^{m_{i}}x_{ij}^{\beta }\ln(x_{ij})-\sum_{i=1}^{k}\frac{m_{i}\psi_{i}^{\prime }(\beta )}{\psi_{i}(\beta )}=0. \end{array}$$ (11)

Equation (11) does not have a closed-form expression. Therefore, numerical methods for solving nonlinear equations, such as the Newton–Raphson method or Brent’s method, are utilized to solve for the MLE $\hat{\beta }$. In the implementation, these numerical procedures were carried out using Wolfram Mathematica (version 14), specifically through the functions FindRoot, NMaximize, and FindMaximum. Once $\hat{\beta }$ is obtained, the corresponding estimates ${\hat{\alpha }}_{i}$ can be evaluated using Equation (10).

By the invariant property, the MLE of the SF and HRF reliability indices evaluated at a fixed time point *x*_0_, are given by


$$\begin{array}{c} \hat{S}(x_{0},\hat{\beta },\hat{\alpha })=e^{\hat{\alpha }(1-e^{x_{0}^{\hat{\beta }}})}\;\text{and}\;\hat{h}(x_{0};\hat{\beta },\hat{\alpha })=\hat{\alpha }\hat{\beta }x_{0}^{\hat{\beta }-1}e^{x_{0}^{\hat{\beta }}}, \end{array}$$

where $\hat{\alpha }=\frac{\sum_{i=1}^{k}\frac{1}{\hat{var}({\hat{\alpha }}_{i})}{\hat{\alpha }}_{i}}{\sum_{i=1}^{k}\frac{1}{\hat{var}({\hat{\alpha }}_{i})}}$. The variance of $\hat{var}({\hat{\alpha }}_{i})$ is discussed in the next section. For a detailed discussion of the invariance property of MLEs and its implications for derived estimators, see Vander and Meeker [24].

The existence and uniqueness of the MLEs of the model parameters are crucial to ensure the validity and interpretability of the estimation process. In this study, we analytically examine the likelihood function associated with the proposed model under the assumed censoring scheme. However, due to the complexity and nonlinearity of the likelihood equations, closed-form solutions for the MLEs are generally not attainable. Instead of pursuing a formal analytical proof, the behavior of the likelihood function is examined numerically. The profile log-likelihood curves, see Figs 5 and 10, exhibit smooth unimodal shapes with a single dominant maximum, indicating that the likelihood surface is well-behaved over the parameter space. These graphical characteristics provide strong evidence that the MLEs exist and are practically unique.

**Fig 3:**
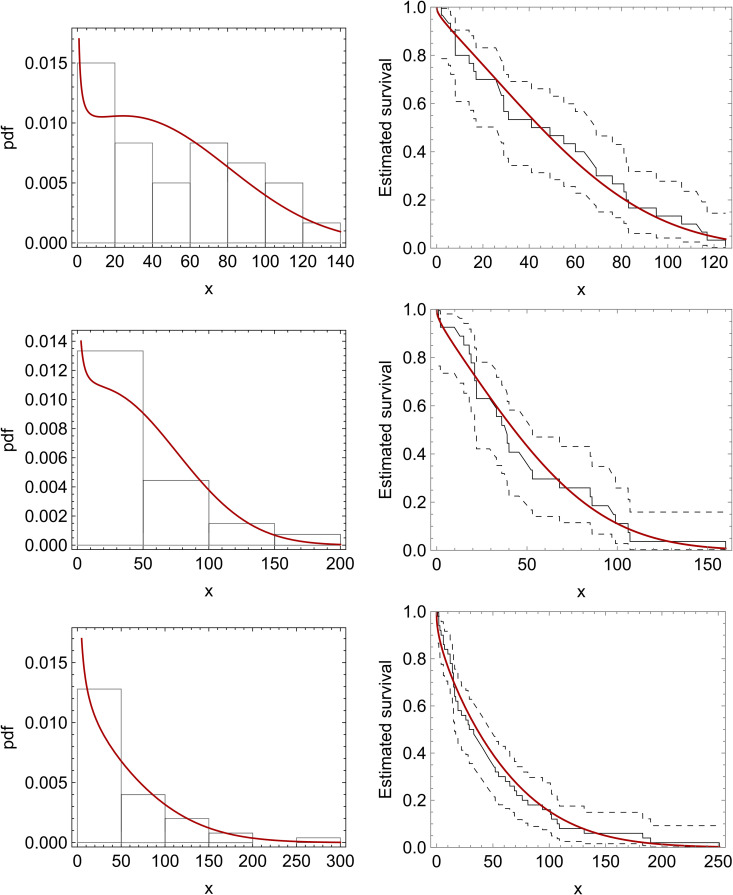
Histogram with estimated PDF and empirical vs theoretical survival functions for the Chen distribution fitted to cancer patients data (from top to bottom: Kidney, Ovary, and Breast).

**Fig 4:**
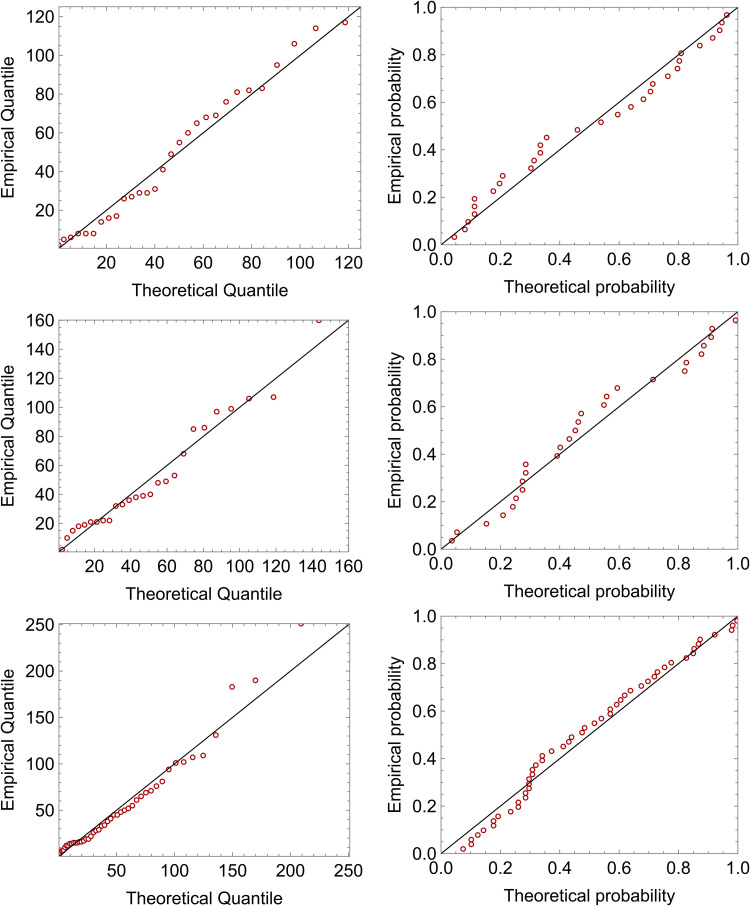
QQ plots and PP plots for the Chen distribution fitted to cancer patients data (from top to bottom: Kidney, Ovary, and Breast).

**Fig 5:**
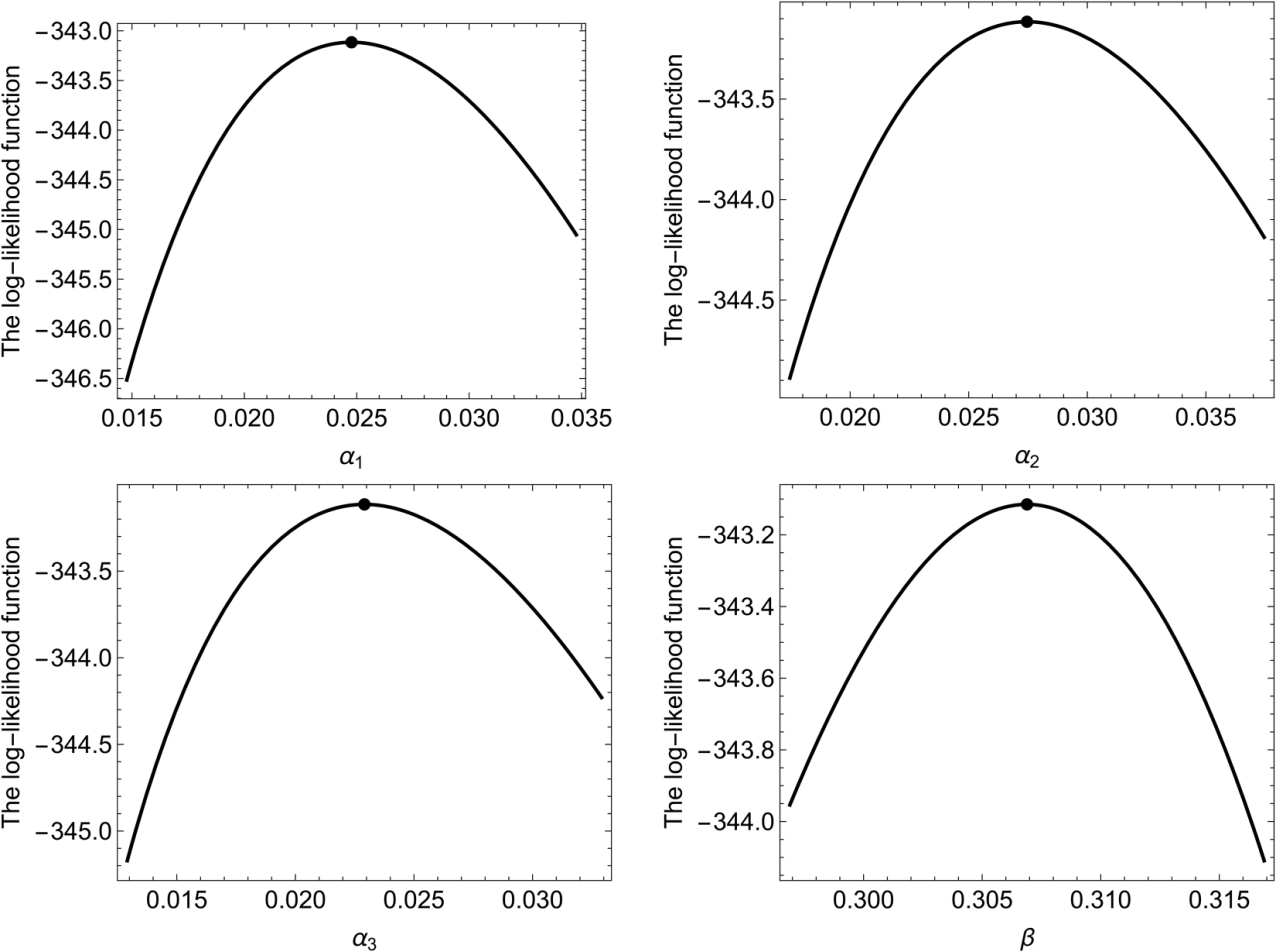
The profile of the log-likelihood function for all parameters for cancer patients data.

**Fig 6:**
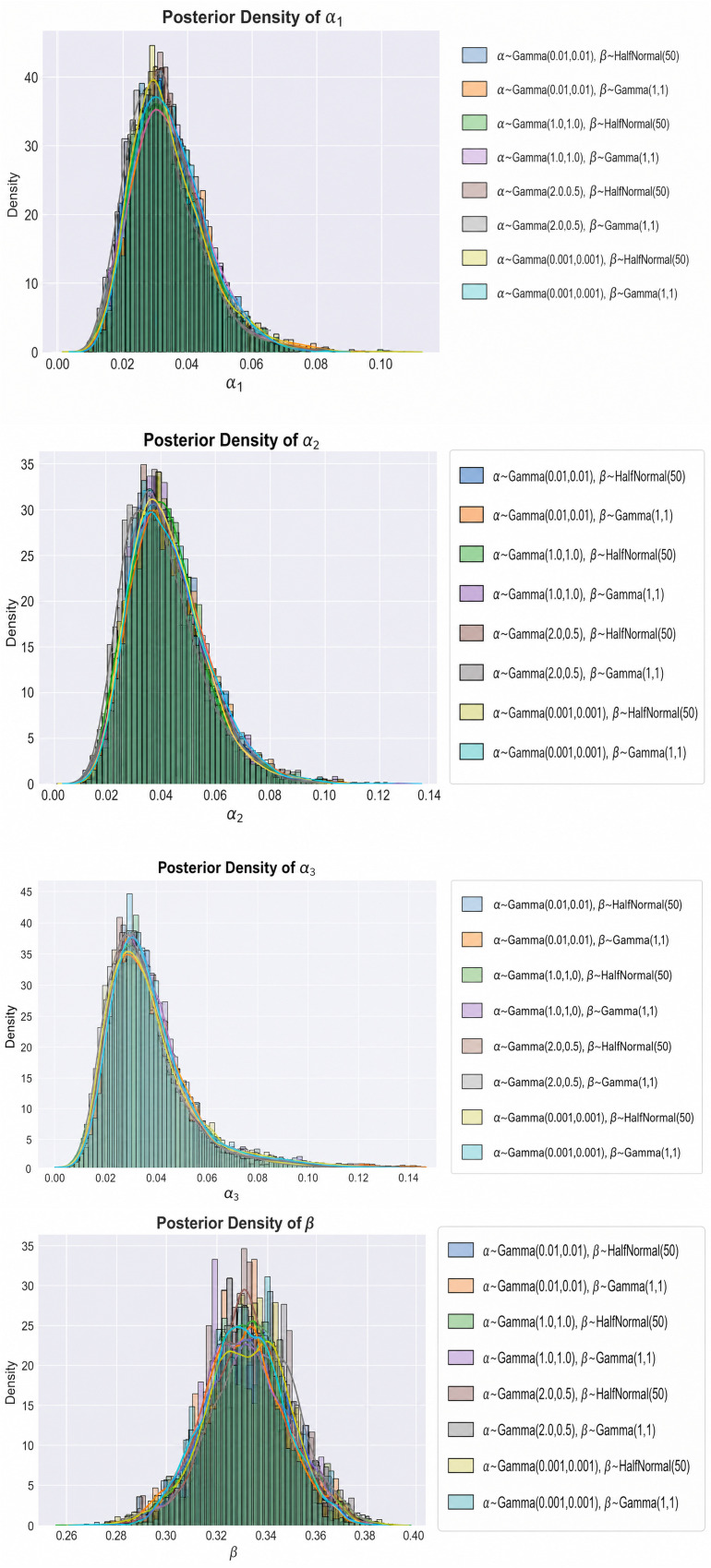
Posterior density for parameters $\alpha_{1},\alpha_{2},\alpha_{3}$ and β under different alternative priors (cancer patients data).

**Fig 7:**
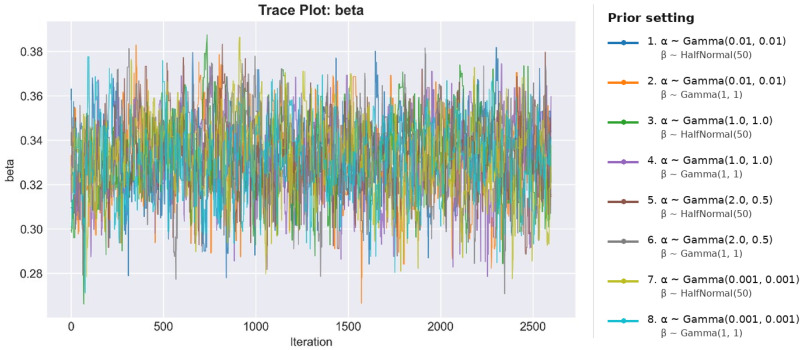
Trace plot for β parameter under different prior alternatives (cancer patients data).

**Fig 8:**
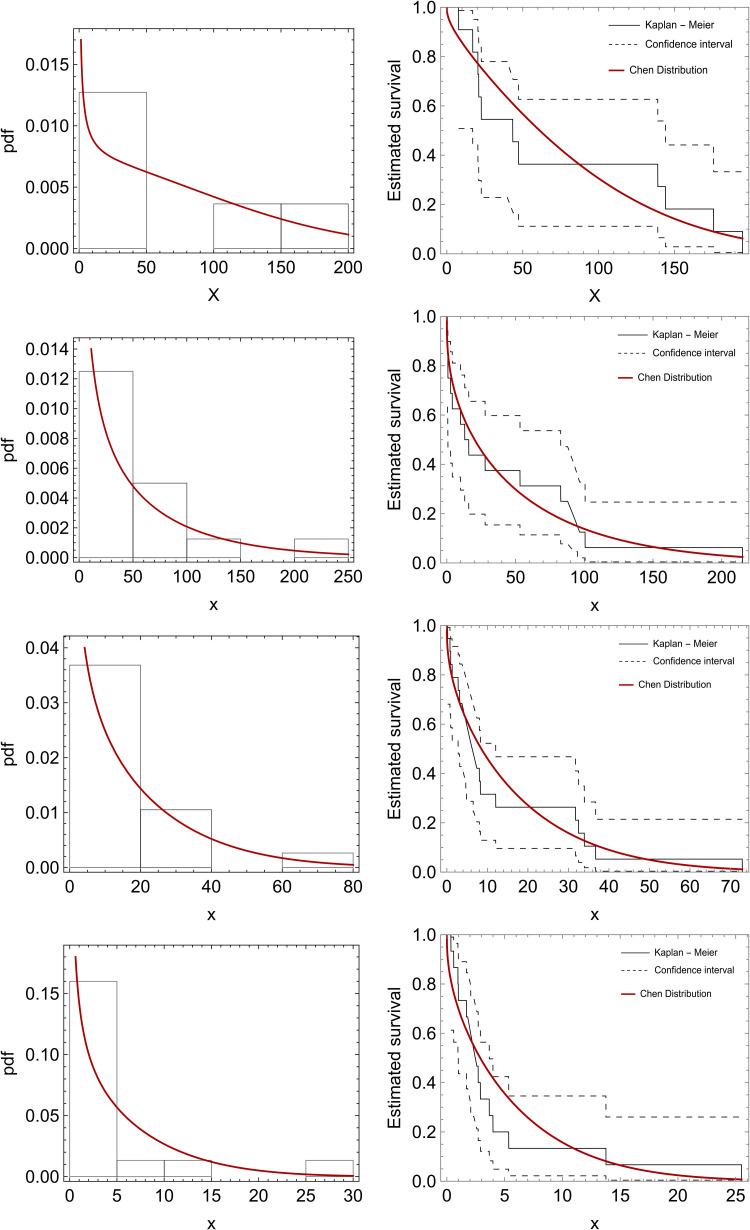
Histogram with estimated PDF and empirical vs theoretical survival functions for the Chen distribution fitted to time to breakdown data (from top to bottom: 30, 32, 34, 36 kV).

**Fig 9:**
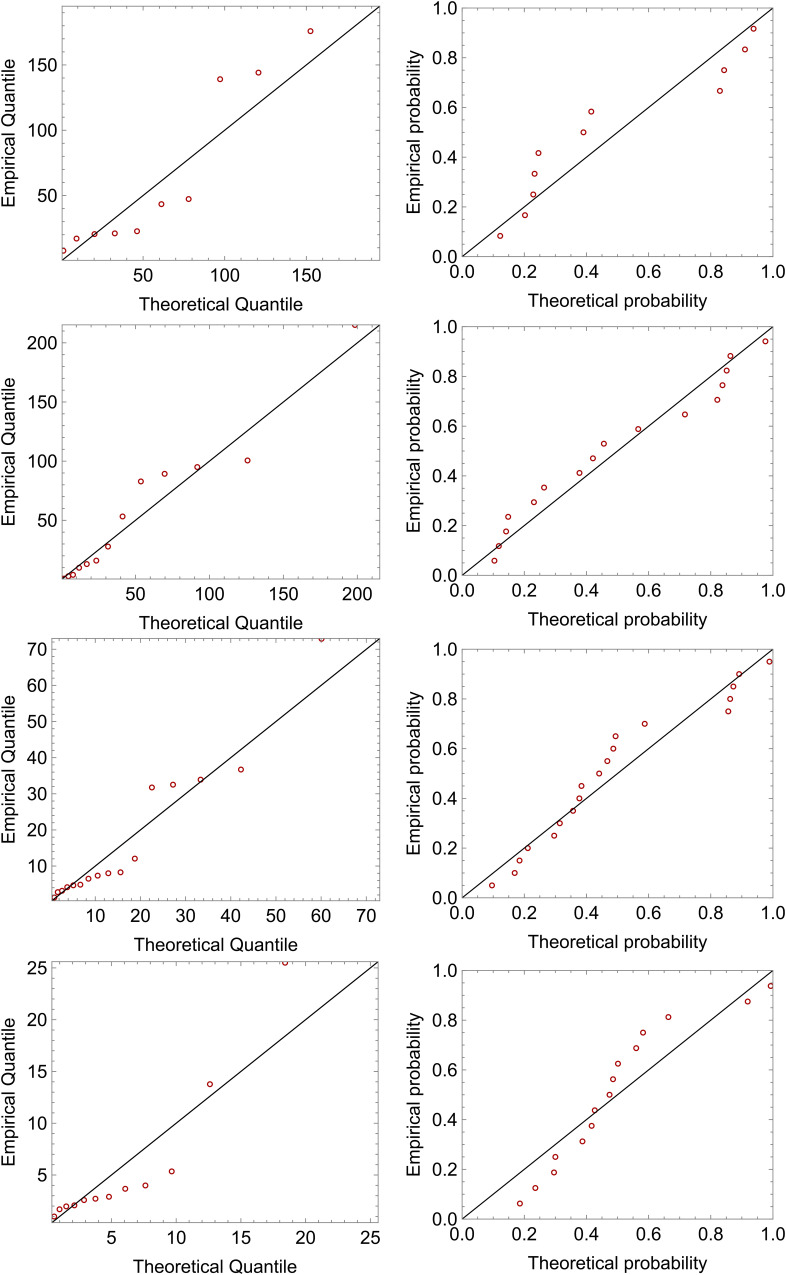
QQ plots, and PP plots for the Chen distribution fitted to time to breakdown data (from top to bottom: 30, 32, 34, 36 kV).

Furthermore, numerical optimization techniques such as the Newton–Raphson and Brent methods are employed to obtain the MLEs. The convergence of these algorithms to the same solution across multiple initial values offers additional numerical support for the stability and consistency of the estimators under different sample sizes and censoring schemes.

### 3.1 Asymptotic confidence intervals

In this section, we examine asymptotic confidence intervals for the unknown parameters and reliability functions associated with the Chen distribution under the BAPCS scheme. Under standard regularity conditions, the maximum likelihood estimators (MLEs) are asymptotically normally distributed. Specifically, the vector of estimators $\left(\hat{\beta },{\hat{\alpha }}_{1},{\hat{\alpha }}_{2},...,{\hat{\alpha }}_{k}\right)$ follows an approximate (*k* + 1)-dimensional multivariate normal distribution. The mean vector of this distribution is the vector of true parameter values, and the associated variance-covariance matrix is the inverse of the Fisher information matrix, denoted by $I^{-1}(\cdot )$. The Fisher information matrix is defined as the expected value of the negative Hessian (i.e., the matrix of second-order partial derivatives) of the log-likelihood function with respect to the parameters $\left(\beta ,\alpha_{1},\alpha_{2},...,\alpha_{k}\right)$. In practice, since the expectations may be analytically intractable, we use the observed Fisher information matrix, which is computed by evaluating the negative second derivatives of the log-likelihood function at the MLEs. This estimated matrix is denoted as $I(\hat{\beta },\hat{\alpha })$ and is given by:


$$\begin{array}{c} I(\hat{\beta },\hat{\alpha })=-[\begin{array}{ccccc} \frac{\partial^{2}\ell }{\partial \beta^{2}} & \frac{\partial^{2}\ell }{\partial \beta \partial \alpha_{1}} & \frac{\partial^{2}\ell }{\partial \beta \partial \alpha_{2}} & ... & \frac{\partial^{2}\ell }{\partial \beta \partial \alpha_{k}}\\ \frac{\partial^{2}\ell }{\partial \alpha_{1}\partial \beta } & \frac{\partial^{2}\ell }{\partial \alpha_{1}^{2}} & \frac{\partial^{2}\ell }{\partial \alpha_{1}\partial \alpha_{2}} & ... & \frac{\partial^{2}\ell }{\partial \alpha_{1}\partial \alpha_{k}}\\ \vdots & \vdots & \vdots & \ddots & \vdots \\ \frac{\partial^{2}\ell }{\partial \alpha_{k}\partial \beta } & \frac{\partial^{2}\ell }{\partial \alpha_{k}\partial \alpha_{1}} & \frac{\partial^{2}\ell }{\partial \alpha_{k}\partial \alpha_{2}} & ... & \frac{\partial^{2}\ell }{\partial \alpha_{k}^{2}} \end{array}], \end{array}$$ (12)

where


$$\begin{array}{c} \frac{\partial^{2}\ell }{\partial \beta^{2}}=\frac{-\sum_{i=1}^{k}m_{i}}{\beta^{2}}+\sum_{i=1}^{k}\sum_{j=1}^{m_{i}}x_{ij}^{\beta }\ln^{2}(x_{ij})-\sum_{i=1}^{k}m_{i}\frac{\psi_{i}(\beta )\psi_{i}^{\prime \prime }(\beta )-(\psi_{i}^{\prime }(\beta ){)}^{2}}{\psi_{i}^{2}(\beta )}, \end{array}$$

$$\begin{array}{c} \frac{\partial^{2}\ell }{\partial \alpha_{i}^{2}}=\frac{-m_{i}}{\alpha_{i}^{2}},\;\frac{\partial^{2}\ell }{\partial \alpha_{i}\partial \alpha_{j}}{|}_{i\neq j}=0,i,j=1,...,k,\;\text{and}\;\frac{\partial^{2}\ell }{\partial \alpha_{i}\partial \beta }=\sum_{i=1}^{k}\psi_{i}^{\prime }(\beta ), \end{array}$$

where


$$\begin{array}{c} \psi_{i}^{\prime }(\beta )=-\sum_{j=1}^{m_{i}}x_{ij}^{\beta }ln(x_{ij})e^{x_{ij}^{\beta }}-\sum_{j=1}^{J_{i}}R_{ij}x_{ij}^{\beta }ln(x_{ij})e^{x_{ij}^{\beta }}-R_{im_{i}}^{*}x_{im_{i}}^{\beta }ln(x_{im_{i}})e^{x_{im_{i}}^{\beta }} \end{array}$$

and


$$\begin{array}{cc} \psi_{i}^{\prime \prime }(\beta ) & =-\sum_{j=1}^{m_{i}}x_{ij}^{\beta }\ln^{2}(x_{ij})e^{x_{ij}^{\beta }}(1+x_{ij}^{\beta })-\sum_{j=1}^{J_{i}}R_{ij}x_{ij}^{\beta }\ln^{2}(x_{ij})e^{x_{ij}^{\beta }}(1+x_{ij}^{\beta })\\ & -R_{im_{i}}^{*}x_{im_{i}}^{\beta }\ln^{2}(x_{im_{i}})e^{x_{im_{i}}^{\beta }}(1+x_{im_{i}}^{\beta }). \end{array}$$

Under mild regularity conditions, the asymptotic distribution of the MLE $\left(\hat{\beta },\hat{\alpha }\right)$, is given by $(\hat{\beta },\hat{\alpha })-(\beta ,\alpha )\to N\left(0,I^{-1}(\hat{\beta },\hat{\alpha })\right)$, where $I^{-1}(\hat{\beta },\hat{\alpha })$ denotes the inverse of the observed Fisher information matrix, defined as follows:


$$\begin{array}{c} I^{-1}(\hat{\beta },\hat{\alpha })=[\begin{array}{ccccc} var(\hat{\beta }) & cov(\hat{\beta },\hat{\alpha_{1}}) & cov(\hat{\beta },\hat{\alpha_{2}}) & ... & cov(\hat{\beta },\hat{\alpha_{k}})\\ cov(\hat{\alpha_{1}},\hat{\beta }) & var(\hat{\alpha_{1}}) & cov(\hat{\alpha_{1}},\hat{\alpha_{2}}) & ... & cov(\hat{\alpha_{1}},\hat{\alpha_{k}})\\ \vdots & \vdots & \vdots & \ddots & \vdots \\ cov(\hat{\alpha_{k}},\hat{\beta }) & cov(\hat{\alpha_{k}},\hat{\alpha_{1}}) & cov(\hat{\alpha_{k}},\hat{\alpha_{2}}) & ... & var(\hat{\alpha_{k}}) \end{array}]. \end{array}$$ (13)

Let $\nu_{ij},\;i,j=1,...,k+1$ denotes the entries of ${\hat{I}}^{-1}(\cdot )$, then $\nu_{11}$ represents the estimated asymptotic variance of $\hat{\beta }$ and $\nu_{ii}$ for i=2,...,k+1 represent the estimated asymptotic variance of $\alpha_{i-1}$. Consequently, the (1−γ)% asymptotic confidence interval ($ACI_{MLE}$) for β is:


$$\begin{array}{c} (\hat{\beta }-z_{\frac{\gamma }{2}}\sqrt{\nu_{11}},\hat{\beta }+z_{\frac{\gamma }{2}}\sqrt{\nu_{11}}), \end{array}$$

and for $\alpha_{i-1},\;i=2,...,k+1$, the corresponding $ACI_{MLE}$ is:


$$\begin{array}{c} ({\hat{\alpha }}_{i-1}-z_{\frac{\gamma }{2}}\sqrt{\nu_{ii}},{\hat{\alpha }}_{i-1}+z_{\frac{\gamma }{2}}\sqrt{\nu_{ii}}), \end{array}$$

where $z_{\frac{\gamma }{2}}$ is defined as $P(Z>z_{\frac{\gamma }{2}})=\frac{\gamma }{2}$, *Z* follows standard normal distribution, i.e., Z~N(0,1).

To construct $ACI_{MLE}$ for nonlinear functions of the model parameters such as the SF, and the HRF, we apply the delta method. For more details on this method, see Beutner et al. [25]. Let ϕ(β,α) be a differentiable function of the parameters, and let $\hat{\phi }=\phi (\hat{\beta },\hat{\alpha })$ denote its MLE. Then, under the asymptotic normality of the MLEs, the approximate variance of $\hat{\phi }$ is given by


$$\begin{array}{c} \hat{var}(\hat{\phi })=[\Delta \phi^{T}(\beta ,\alpha )]I^{-1}(\hat{\beta },\hat{\alpha })[\Delta \phi (\beta ,\alpha )], \end{array}$$ (14)

where $\Delta \phi^{T}$ denotes the gradient vector of partial derivatives with respect to the parameters and given by


$$\begin{array}{c} \Delta \phi^{T}(\beta ,\alpha )={\left(\frac{\partial \phi }{\partial \beta },\frac{\partial \phi }{\partial \alpha_{1}},...,\frac{\partial \phi }{\partial \alpha_{k}}\right)}^{T}{|}_{\left(\beta ,\alpha )=(\hat{\beta },\hat{\alpha }\right)}. \end{array}$$

According to the delta method, the asymptotic distribution of ϕ satisfies:


$$\begin{array}{c} \frac{\hat{\phi }-\phi }{\sqrt{\hat{var}(\hat{\phi })}}\to N(0,1), \end{array}$$

Consequently, the 100(1−γ)% $ACI_{MLE}$ for ϕ(β,α) can be derived as


$$\begin{array}{c} \left(\hat{\phi }-z_{\frac{\gamma }{2}}\sqrt{\hat{var}(\hat{\phi }})\;,\;\hat{\phi }+z_{\frac{\gamma }{2}}\sqrt{\hat{var}(\hat{\phi })}\right) \end{array}$$

As the lower bound of the $ACI_{MLE}$ could be less than zero, while the parameters β and $\alpha_{i}$ (i=1,...,k) as well as the reliability measures *S*(*x*) and *h*(*x*) are restricted to the positive domain; an adjustment to the interval estimates is necessary. To maintain consistency with this constraint, a modified confidence interval is adopted for any positive quantity $\theta \in \{\beta ,\alpha_{1},...,\alpha_{k},\phi (\beta ,\alpha )\}$, defined as


$$\begin{array}{c} \left(\max\left[0,\;\hat{\theta }-z_{\gamma /2}\sqrt{\hat{var}(\hat{\theta })}\right],\;\;\hat{\theta }+z_{\gamma /2}\sqrt{\hat{var}(\hat{\theta })}\right). \end{array}$$

## 4. Bayesian estimation

This section introduces a hierarchical Bayesian method for estimating the model’s parameters and reliability functions. Furthermore, it is assumed that $\alpha_{i}$, for *i* = 1, 2, ..., *k*, follows a shared independent gamma prior to hyperparameters η and ξ. The Gamma distribution was selected as the prior for $\alpha_{i}$ because it is the conjugate prior for the scale parameter in many lifetime and reliability models. Using a conjugate prior simplifies the posterior derivation and ensures analytical tractability, which is particularly useful for the Bayesian framework. Moreover, the Gamma distribution is flexible in modeling positive continuous parameters, and it can represent a wide range of prior beliefs, from weakly informative to highly concentrated. In our context, $\alpha_{i}$ is strictly positive and governs the shape of the underlying hazard structure; thus, the Gamma prior naturally encodes this positivity constraint while allowing incorporation of prior knowledge from historical data and previous studies.

As a result, the joint prior of $\left(\alpha_{1},\alpha_{2},...,\alpha_{k}\right)$ can be written as


$$\begin{array}{c} \pi (\alpha_{1},\alpha_{2},...,\alpha_{k}|\eta ,\xi )\propto {\left(\prod_{i=1}^{k}\alpha_{i}\right)}^{\eta -1}e^{-\xi \sum_{i=1}^{k}\alpha_{i}}\eta ,\xi ,\alpha_{i}>0. \end{array}$$

In this framework, the variance term $\eta /\xi^{2}$ associated with $\alpha_{i}$ captures the degree of variability between the facilities and quantifies the extent of the differential degree of failure (DDF). To maintain generality and avoid imposing strong assumptions, non-informative priors are assigned to the second-stage hyperparameters η and ξ. Furthermore, a joint prior distribution is specified for the pair (η,ξ), as defined by


$$\begin{array}{c} \pi (\eta ,\xi )\propto 1,\eta ,\xi >0. \end{array}$$

Additionally, the parameter β is assigned an independent non-informative prior, specified as


$$\begin{array}{c} \pi (\beta )\propto 1,\beta >0. \end{array}$$

Therefore, the joint posterior distribution of $\tau =(\beta ,\alpha_{1},\alpha_{2},...,\alpha_{k},\eta ,\xi )$ given the BAPCS data $\vartheta^{\prime }$ can be expressed as


$$\begin{array}{c} \pi (\tau |\vartheta^{\prime })\propto \pi (\beta )\times \pi (\eta ,\xi )\times \pi (\alpha_{1},\alpha_{2},...,\alpha_{k}|\eta ,\xi )\times L(\beta ,\alpha ,\vartheta^{\prime }) \end{array}$$

$$\begin{array}{c} \propto \beta^{\sum_{i=1}^{k}m_{i}}\prod_{i=1}^{k}\alpha_{i}^{m_{i}+\eta -1}\prod_{i=1}^{k}\prod_{j=1}^{m_{i}}x_{ij}^{\beta -1}e^{\sum_{j=1}^{m_{i}}\sum_{i=1}^{k}x_{ij}^{\beta }}e^{\sum_{i=1}^{k}\alpha_{i}(-\xi +\psi_{i}(\beta ))}. \end{array}$$ (15)

Within the Bayesian framework, when the squared error loss function is adopted as the criterion for estimation, the optimal Bayesian estimator of the quantity M(τ), which is a function dependent on the unknown parameter τ, is obtained by computing the posterior expectation of M(τ) with respect to the posterior distribution of τ. Mathematically, this estimator is expressed as


$$\begin{array}{c} \hat{M}(\tau )=\frac{\oint M(\tau )\pi (\beta )\pi (\eta ,\xi )\pi (\alpha |\eta ,\xi )L(\beta ,\alpha ,\vartheta^{\prime })d\tau }{\oint \pi (\beta )\pi (\eta ,\xi )\pi (\alpha |\eta ,\xi )L(\beta ,\alpha ,\vartheta^{\prime })d\tau }. \end{array}$$ (16)

It is evident that the ratio of the two integrals in the Bayesian estimator of M(τ) does not yield a closed-form solution, making analytical evaluation intractable. To overcome this limitation and to obtain both the Bayes estimates and the Highest Posterior Density (HPD) credible interval for M(τ), we employ a Markov Chain Monte Carlo (MCMC) simulation method. This computational approach is implemented within a hierarchical Bayesian framework, which enables efficient approximation of the posterior quantities of interest. The detailed structure and steps of the MCMC procedure are presented in the following section.

### 4.1 Analysis of the posterior distribution and MCMC sampling

The Gibbs sampling technique is an effective method for generating a Markov chain from a posterior distribution based on the observed data. This sampling method simplifies the process of handling complex posterior computations. Using the posterior distribution in Equation (15), the conditional posterior density of $\alpha_{i}$, where *i* = 1, 2, ..., *k*, can be expressed as follows:


$$\begin{array}{c} \pi (\alpha_{i}|\beta ,\eta ,\xi ;\vartheta_{i}^{\prime })\propto \prod_{i=1}^{k}\alpha_{i}^{m_{i}+\eta -1}e^{\sum_{i=1}^{k}\alpha_{i}(-\xi +\psi_{i}(\beta ))}. \end{array}$$ (17)

Furthermore, the conditional posterior densities of η and ξ are expressed as follows:


$$\begin{array}{c} \pi (\eta |\alpha )\propto {\left(\prod_{i=1}^{k}\alpha_{i}\right)}^{\eta -1} \end{array}$$ (18)

and


$$\begin{array}{c} \pi (\xi |\alpha )\propto e^{-\sum_{i=1}^{k}\xi \alpha_{i}}. \end{array}$$ (19)

In addition, the conditional density of β is obtained as follows:


$$\begin{array}{c} \pi (\beta |\alpha ,\xi ;\vartheta^{\prime })\propto \beta^{\sum_{i=1}^{k}m_{i}}\prod_{i=1}^{k}\prod_{j=1}^{m_{i}}x_{ij}^{\beta -1}e^{\sum_{j=1}^{m_{i}}\sum_{i=1}^{k}x_{ij}^{\beta }}e^{\sum_{i=1}^{k}\alpha_{i}\psi_{i}(\beta )}. \end{array}$$ (20)

As shown in Equation (17), both $\left(m_{i}+\eta \right)$ and $\left(\xi +\psi_{i}(\beta )\right)$ are strictly positive, implying that the posterior distribution of $\alpha_{i}$ (for *i* = 1, 2, ..., *k*) follows a Gamma distribution with shape parameter $\left(m_{i}+\eta \right)$ and rate parameter $\left(\xi +\psi_{i}(\beta )\right)$. Furthermore, the posterior distribution of ξ, as derived in Equation (19), corresponds to an exponential distribution with parameter $\sum_{i=1}^{k}\alpha_{i}$.

However, the conditional posterior distributions presented in Equations (18) and (20) do not simplify into standard distributions, making the direct sampling from these posteriors analytically intractable. To address this challenge, we employ the Metropolis-Hastings (MH) algorithm, utilizing a normal proposal distribution to generate posterior samples. This approach allows for efficient approximation of the posterior distributions despite their complex forms. The full implementation details of the Metropolis–Hastings algorithm is outlined in [21].

### 4.2 Sensitivity analysis

To assess the robustness of the Bayesian inference to prior specification, we conducted a comprehensive sensitivity analysis by varying the hyperparameters η and ξ of the Gamma priors on $\alpha_{i}$. Four alternative prior specifications are considered for each $\alpha_{i}$:


$$\begin{array}{c} \alpha_{i}\sim \text{Gamma}(0.01,0.01),\;\alpha_{i}\sim \text{Gamma}(1,1), \end{array}$$

$$\begin{array}{c} \alpha_{i}\sim \text{Gamma}(2,0.5),\;\alpha_{i}\sim \text{Gamma}(0.001,0.001), \end{array}$$

representing extremely diffuse, weakly informative, and moderately informative prior beliefs. For the common parameter β, we examined both a diffuse Half–Normal prior,


$$\begin{array}{c} \beta \sim \text{HalfNormal}(50), \end{array}$$

and the original prior based on


$$\begin{array}{c} \beta \sim \text{Gamma}(1,1). \end{array}$$

For each prior combination, posterior inference was obtained using MCMC sampling under the complete BAPCS likelihood. The analysis was carried out using the real failure-time observations given in section 7 for the two testing facilities. The likelihood component incorporated both the failure times and the removal counts generated by the BAPCS mechanism, ensuring that the Bayesian posterior reflects the full censoring structure of the experiment.

Across all prior settings, the posterior density estimates for β and $\alpha_{i}$ are highly stable. The resulting posterior curves exhibited substantial overlap, and robustness is observed for the facility parameters.

A graphical comparison of the posterior densities under the four prior specifications in addition to sensitivity metrics are explored in details in the application part in Section 7. Overall, the sensitivity analysis demonstrates that the proposed Bayesian procedure exhibits strong stability with respect to the prior choices for both β and $\alpha_{i}$. Consequently, the inferential conclusions drawn in the Bayesian section are not artifacts of prior selection but are instead well supported by the observed BAPCS data.

## 5. Maximum product of spacings estimation

The maximum product of spacings estimation (MPSE) method is a robust alternative to the MLE, particularly in situations where the likelihood function is unbounded or when data arise from heavy-tailed distributions. MPSE produces consistent and asymptotically efficient estimators and is widely considered a reliable technique for estimating parameters in complex statistical models. MPSE has been effectively utilized under various censoring schemes, including APCS, as demonstrated in several recent studies [6,26–28].

Based on the observed sample for the $i^{th}$ facility under BAPCS in Table 1, the product of spacings function for the $i^{th}$ facility is defined as


$$\begin{array}{c} D_{i}(\alpha_{i},\beta )=\prod_{j=1}^{m_{i}+1}\left[F(x_{ij};\alpha_{i},\beta )-F(x_{ij-1};\alpha_{i},\beta )\right]\prod_{j=1}^{J_{i}}{\left[1-F(x_{ij};\alpha_{i},\beta )\right]}^{R_{ij}}\times [1-F(x_{im_{i}};\alpha_{i},\beta ){]}^{R_{im_{i}}^{*}}, \end{array}$$

where $F(x_{i0};\alpha_{i},\beta )=0$, $F(x_{im_{i}+1};\alpha_{i},\beta )=1$ and $\sum_{j=1}^{m_{i}+1}D_{ij}(\alpha_{i},\beta )=1$. Then, the product of spacings for the entire BAPCS setup is expressed as


$$\begin{array}{c} D(\alpha_{i},\beta )=\prod_{i=1}^{k}\prod_{j=1}^{m_{i}+1}\left[F(x_{ij};\alpha_{i},\beta )-F(x_{ij-1};\alpha_{i},\beta )\right]\prod_{j=1}^{J_{i}}{\left[1-F(x_{ij};\alpha_{i},\beta )\right]}^{R_{ij}}\times [1-F(x_{im_{i}};\alpha_{i},\beta ){]}^{R_{im_{i}}^{*}}. \end{array}$$

Substituting the Chen CDF from Equation (4), the product of spacings simplifies to


$$\begin{array}{c} D(\alpha_{i},\beta )=\prod_{i=1}^{k}\prod_{j=1}^{m_{i}+1}\left[e^{\alpha_{i}(1-e^{x_{ij-1}^{\beta }})}-e^{\alpha_{i}(1-e^{x_{ij}^{\beta }})}\right]\prod_{j=1}^{J_{i}}{\left[e^{\alpha_{i}(1-e^{x_{ij}^{\beta }})}\right]}^{R_{ij}}\times [e^{\alpha_{i}(1-e^{x_{im_{i}}^{\beta }})}{]}^{R_{im_{i}}^{*}}. \end{array}$$

The MPSE of β and $\alpha_{i}$ are obtained by maximizing the logarithmic of the product of spacings:


$$\begin{array}{cc} \ell_{MPS}=\ln D(\alpha_{i},\beta ) & =\sum_{i=1}^{k}\sum_{j=1}^{m_{i}}\ln\left[e^{\alpha_{i}(1-e^{x_{ij-1}^{\beta }})}-e^{\alpha_{i}(1-e^{x_{ij}^{\beta }})}\right]\\ & +\sum_{i=1}^{k}\sum_{j=1}^{J_{i}}R_{ij}\alpha_{i}(1-e^{x_{ij}^{\beta }})+\sum_{i=1}^{k}R_{im_{i}}^{*}\alpha_{i}(1-e^{x_{im_{i}}^{\beta }}). \end{array}$$ (21)

The MPSE of β and $\alpha_{i}$, denoted as $\tilde{\beta }$ and $\tilde{\alpha_{i}}$, are obtained by solving the following nonlinear system:


$$\begin{array}{cc} \frac{\partial \ell_{MPS}}{\partial \beta } & =\sum_{i=1}^{k}\sum_{j=1}^{m_{i}}\frac{\Upsilon_{ij}-\Upsilon_{ij-1}}{e^{\alpha_{i}(1-e^{x_{ij-1}^{\beta }})}-e^{\alpha_{i}(1-e^{x_{ij}^{\beta }})}}-\sum_{i=1}^{k}\sum_{j=1}^{J_{i}}R_{ij}\alpha_{i}x_{ij}^{\beta }\ln(x_{ij})e^{x_{ij}^{\beta }}\\ & -\sum_{i=1}^{k}R_{im_{i}}^{*}\alpha_{i}x_{im_{i}}^{\beta }\ln(x_{im_{i}})e^{x_{im_{i}}^{\beta }}=0 \end{array}$$ (22)

$$\begin{array}{cc} \frac{\partial \ell_{MPS}}{\partial \alpha_{i}} & =\sum_{i=1}^{k}\sum_{j=1}^{m_{i}}\frac{\Psi_{ij-1}-\Psi_{ij}}{e^{\alpha_{i}(1-e^{x_{ij-1}^{\beta }})}-e^{\alpha_{i}(1-e^{x_{ij}^{\beta }})}}+\sum_{i=1}^{k}\sum_{j=1}^{J_{i}}R_{ij}(1-e^{x_{ij}^{\beta }})\\ & +\sum_{i=1}^{k}R_{im_{i}}^{*}(1-e^{x_{im_{i}}^{\beta }})=0, \end{array}$$ (23)

where $\Upsilon_{ij}=\alpha_{i}x_{ij}^{\beta }\ln(x_{ij})e^{\alpha_{i}(1-e^{x_{ij}^{\beta }})+x_{ij}^{\beta }}$ and $\Psi_{ij}=(1-e^{x_{ij-1}^{\beta }})e^{\alpha_{i}(1-e^{x_{ij-1}^{\beta }})}$. This system must be solved numerically, as it does not admit closed-form solutions. It is important to note that MPSE can be sensitive to data with closely spaced or tied observations. In such cases, where the spacing between ordered data points is zero, it is recommended to replace the zero spacing with the corresponding likelihood contribution to maintain numerical stability. The MPSE of the SF and HRF are evaluated at a fixed time point *x*_0_ as


$$\begin{array}{c} \tilde{S}(x_{0},\tilde{\beta },\tilde{\alpha })=e^{\tilde{\alpha }(1-e^{x_{0}^{\tilde{\beta }}})}\;\text{and}\;\tilde{h}(x_{0};\tilde{\beta },\tilde{\alpha })=\tilde{\alpha }\tilde{\beta }x_{0}^{\tilde{\beta }-1}e^{x_{0}^{\tilde{\beta }}}, \end{array}$$

where $\tilde{\alpha }=\frac{\sum_{i=1}^{k}\frac{1}{\tilde{var}({\tilde{\alpha }}_{i})}{\tilde{\alpha }}_{i}}{\sum_{i=1}^{k}\frac{1}{\tilde{var}({\tilde{\alpha }}_{i})}}$. The variance of $\tilde{var}({\tilde{\alpha }}_{i})$ is discussed in the next section.

### 5.1 Asymptotic confidence intervals

The asymptotic confidence interval based on MPSE ($ACI_{MPSE}$) is constructed using the well-established asymptotic equivalence between MLE and MPSE, as discused in [29–31]. In this context, the asymptotic variance-covariance matrix of the MPSEs is given by


$$\begin{array}{c} I_{MPS}^{-1}(\tilde{\beta },\tilde{\alpha })=[\begin{array}{ccccc} -\frac{\partial^{2}\ell_{MPS}}{\partial \beta^{2}} & -\frac{\partial^{2}\ell_{MPS}}{\partial \beta \partial \alpha_{1}} & -\frac{\partial^{2}\ell_{MPS}}{\partial \beta \partial \alpha_{2}} & ... & -\frac{\partial^{2}\ell_{MPS}}{\partial \beta \partial \alpha_{k}}\\ -\frac{\partial^{2}\ell_{MPS}}{\partial \alpha_{1}\partial \beta } & -\frac{\partial^{2}\ell_{MPS}}{\partial \alpha_{1}^{2}} & -\frac{\partial^{2}\ell_{MPS}}{\partial \alpha_{1}\partial \alpha_{2}} & ... & -\frac{\partial^{2}\ell_{MPS}}{\partial \alpha_{1}\partial \alpha_{k}}\\ \vdots & \vdots & \vdots & \ddots & \vdots \\ -\frac{\partial^{2}\ell_{MPS}}{\partial \alpha_{k}\partial \beta } & -\frac{\partial^{2}\ell_{MPS}}{\partial \alpha_{k}\partial \alpha_{1}} & -\frac{\partial^{2}\ell_{MPS}}{\partial \alpha_{k}\partial \alpha_{2}} & ... & -\frac{\partial^{2}\ell_{MPS}}{\partial \alpha_{k}^{2}} \end{array}{]}_{\left(\beta ,\alpha )=(\tilde{\beta },\tilde{\alpha }\right)}^{-1}, \end{array}$$ (24)

where $\tilde{\alpha }=(\tilde{\alpha_{1}},\tilde{\alpha_{2}},...,\tilde{\alpha_{k}})$ and


$$\begin{array}{cc} \frac{\partial^{2}\ell_{MPS}}{\partial \beta^{2}} & =\sum_{i=1}^{k}\sum_{j=1}^{J_{i}}\frac{\Upsilon_{ij}^{\prime }-\Upsilon_{ij-1}^{\prime }}{e^{\alpha_{i}(1-e^{x_{ij-1}^{\beta }})}-e^{\alpha_{i}(1-e^{x_{ij}^{\beta }})}}-\sum_{i=1}^{k}\sum_{j=1}^{J_{i}}\frac{(\Upsilon_{ij}-\Upsilon_{ij-1}{)}^{2}}{{\left(e^{\alpha_{i}(1-e^{x_{ij-1}^{\beta }})}-e^{\alpha_{i}(1-e^{x_{ij}^{\beta }})}\right)}^{2}}\\ & -\sum_{i=1}^{k}\sum_{j=1}^{J_{i}}R_{ij}\alpha_{i}x_{ij}^{\beta }(1+x_{ij}^{\beta })\ln^{2}(x_{ij})e^{x_{ij}^{\beta }}-\sum_{i=1}^{k}R_{im_{i}}^{*}\alpha_{i}x_{im_{i}}^{\beta }(1+x_{im_{i}}^{\beta })\ln^{2}(x_{im_{i}})e^{x_{im_{i}}^{\beta }} \end{array}$$

$$\begin{array}{c} \frac{\partial^{2}\ell_{MPS}}{\partial \alpha_{i}^{2}}=-\sum_{i=1}^{k}\sum_{j=1}^{m_{i}}\frac{{\left(e^{x_{ij-1}^{\beta }}-e^{x_{ij}^{\beta }}\right)}^{2}e^{\alpha_{i}\left(e^{x_{ij-1}^{\beta }}+e^{x_{ij}^{\beta }}\right)}}{{\left(e^{\alpha_{i}e^{x_{ij-1}^{\beta }}}-e^{\alpha_{i}e^{x_{ij}^{\beta }}}\right)}^{2}},\;\frac{\partial^{2}\ell_{MPS}}{\partial \alpha_{i}\partial \alpha_{j}}{|}_{i\neq j}=0,i,j=1,...,k, \end{array}$$

$$\begin{array}{cc} \frac{\partial^{2}\ell_{MPS}}{\partial \alpha_{i}\beta } & =\sum_{i=1}^{k}\sum_{j=1}^{J_{i}}\frac{\Psi_{ij-1}^{\prime }-\Psi_{ij}^{\prime }}{e^{\alpha_{i}(1-e^{x_{ij-1}^{\beta }})}-e^{\alpha_{i}(1-e^{x_{ij}^{\beta }})}}-\sum_{i=1}^{k}\sum_{j=1}^{J_{i}}\frac{(\Psi_{ij-1}-\Psi_{ij}{)}^{2}}{{\left(e^{\alpha_{i}(1-e^{x_{ij-1}^{\beta }})}-e^{\alpha_{i}(1-e^{x_{ij}^{\beta }})}\right)}^{2}}\\ & -\sum_{i=1}^{k}\sum_{j=1}^{J_{i}}R_{ij}x_{ij}^{\beta }\ln\left(x_{ij}\right)e^{x_{ij}^{\beta }}-\sum_{i=1}^{k}R_{im_{i}}^{*}\alpha_{i}x_{im_{i}}^{\beta }\ln(x_{im_{i}})e^{x_{im_{i}}^{\beta }}, \end{array}$$

where


$$\begin{array}{c} \Upsilon_{ij}^{\prime }=\alpha_{i}x_{ij}^{\beta }\ln^{2}\left(x_{ij}\right)e^{\alpha_{i}-\alpha_{i}e^{x_{ij}^{\beta }}+x_{ij}^{\beta }}\left(x_{ij}^{\beta }\left(\alpha_{i}e^{x_{ij}^{\beta }}-1\right)-1\right), \end{array}$$

and


$$\begin{array}{c} \Psi_{ij}^{\prime }=x_{ij}^{\beta }\log\left(x_{ij}\right)e^{\alpha_{i}-\alpha_{i}e^{x_{ij}^{\beta }}+x_{ij}^{\beta }}\left(\alpha_{i}\left(e^{x_{ij}^{\beta }}-1\right)-1\right). \end{array}$$

Thus, the 100(1−γ)% $ACI_{MPSE}$ for the parameters are given by


$$\begin{array}{c} \left(\tilde{\beta }-z_{\frac{\gamma }{2}}\sqrt{var(\tilde{\beta })},\tilde{\beta }+z_{\frac{\gamma }{2}}\sqrt{var(\tilde{\beta })}\right), \end{array}$$

and


$$\begin{array}{c} \left(\tilde{\alpha_{i}}-z_{\frac{\gamma }{2}}\sqrt{var(\tilde{\alpha_{i}})},\tilde{\alpha_{i}}+z_{\frac{\gamma }{2}}\sqrt{var(\tilde{\alpha_{i}})}\right), \end{array}$$

where $var(\tilde{\beta })$ and $var(\tilde{\alpha_{i}})$ denote the asymptotic variances obtained from the diagonal elements of $I_{MPS}^{-1}(\tilde{\beta },\tilde{\alpha })$.

For any differentiable function ϕ(β,α), the asymptotic variance of its MPSE, $\tilde{\phi }$, is


$$\begin{array}{c} \tilde{var}(\tilde{\phi })=[\Delta \phi^{T}(\beta ,\alpha )]I_{MPS}^{-1}(\tilde{\beta },\tilde{\alpha })[\Delta \phi (\beta ,\alpha )]. \end{array}$$ (25)

According to delta method, the 100(1−γ)% $ACI_{MPS}$ for ϕ(β,α) is


$$\begin{array}{c} \left(\tilde{\phi }-z_{\frac{\gamma }{2}}\sqrt{\tilde{var}(\hat{\phi }})\;,\;\tilde{\phi }+z_{\frac{\gamma }{2}}\sqrt{\tilde{var}(\tilde{\phi })}\right). \end{array}$$

As the parameters and the reliability indices are restricted to the positive domain, the lower bound of $ACI_{MPS}$ is truncated at 0 whenever it becomes negative.

## 6. Simulation study

A comprehensive simulation study is conducted to evaluate the performance of the proposed estimators derived using the maximum likelihood estimate, maximum product spacings, and Bayesian methods. The study focuses on estimating the parameters and reliability indices of the Chen distribution under the BAPCS scheme.

To assess the precision and efficiency of each estimation method, four standard performance metrics are used: Mean Squared Error (MSE), average bias (AB), average width (AW) of the confidence interval, and the coverage probability (CP). These metrics are widely used in the literature to evaluate the consistency and reliability of estimators, particularly under complex censoring schemes such as BAPCS. The MSE and AB are used to assess the precision of point estimation to determine how closely the estimators converge to the true parameter values, while interval estimates are assessed using AW and CP. All interval estimates are constructed at the confidence level 95% (for frequentist methods) or the credibility level 95% (for Bayesian methods).

The simulation considers multiple scenarios by varying the number of test facilities *k* and the corresponding effective sample sizes $\left(n_{i},m_{i}\right)$, for i=1,...,k. To realistically reflect heterogeneity across testing environments, a random noise component is introduced for each facility. This variability is modeled using a normally distributed random term with mean zero and a small variance, specifically *N*(0,0.0001), to simulate minor fluctuations in failure times between facilities.

To generate BAPCS data from the Chen distribution, we set the true parameter values as (α,β)=(1.5,0.5). We consider two cases: one with *k* = 3 groups and another with *k* = 4 groups, where each group is subject to a predetermined time duration $T_{i}$, for i=1,...,k. In addition, different censoring schemes are explored across multiple testing facilities, as detailed below.

- **CS-I:**

$$\begin{array}{c} R_{i}=\left((n_{i}-m_{i}),0^{*(m_{i}-1)}\right),\;\text{for }i=1,2,...,k. \end{array}$$

- **CS-II:** For *k* = 3,

$$\begin{array}{c} \begin{array}{cc} R_{1} & =\left((n_{1}-m_{1}),0^{*(m_{1}-1)}\right),\\ R_{2} & =\left(1^{*(n_{2}-m_{2})},0^{*(2m_{2}-n_{2})}\right),\\ R_{3} & =\left(0^{*(2m_{3}-n_{3})},1^{*(n_{3}-m_{3})}\right). \end{array} \end{array}$$

- **CS-III:** For *k* = 4,

$$\begin{array}{c} \begin{array}{cc} R_{1} & =\left((n_{1}-m_{1}),0^{*(m_{1}-1)}\right),\\ R_{2} & =\left(1^{*(n_{2}-m_{2})},0^{*(2m_{2}-n_{2})}\right),\\ R_{3} & =\left(0^{*(2m_{3}-n_{3})},1^{*(n_{3}-m_{3})}\right),\\ R_{4} & =\left((n_{4}-m_{4}),0^{*(m_{4}-1)}\right), \end{array} \end{array}$$

where the notation $a^{*c}$ denotes that the value *a* appears consecutively *c* times in the given scheme. Table 3 summarizes the different scenarios considered in this simulation. A number of 1000 simulated data are generated following the algorithm proposed in [2], with detailed steps outlined as follows:

1. Set the number of groups *k* and determine the values of $n_{i},m_{i},R_{i}$ and $T_{i}$ for i=1,...,k, along with the true values of the parameters (α,β).
2. For each group i=1,...,k, generate $m_{i}$ independent random variables $W_{i1},W_{i2},...,W_{im_{i}}$ from a uniform distribution *U*(0, 1).
3. Compute $V_{il}=W_{il}^{1/(l+\sum_{j=m_{i}-l+1}^{m_{i}}R_{ij})},\;\text{for }l=1,2,...,m_{i}.$
4. Define $U_{il}=1-V_{i(m_{i})}V_{i(m_{i}-1)}\cdots V_{i(m_{i}-l+1)}$ for $l=1,2,...,m_{i}$, yielding *k* groups of PTIIC samples of sizes $m_{i}$ from the *U*(0, 1) distribution, denoted as $U_{i1},U_{i2},...,U_{im_{i}}$.
5. For each group *i*, determine $J_{i}$ such that $Y_{i(J_{i}:m_{i}:n_{i})}<T_{i}<Y_{i(J_{i}+1:m_{i}:n_{i})}$ and discard the samples $Y_{i(J_{i}+2:m_{i}:n_{i})},...,Y_{i(m_{i}:m_{i}:n_{i})}$.
6. Generate the first $m_{i}-J_{i}-1$ order statistics from the truncated distribution $\frac{f(x_{i})}{1-F(x_{i(J_{i}+1:m_{i}:n_{i})})}$ with sample size $n_{i}-\sum_{l=1}^{J_{i}}R_{il}-J_{i}-1$, denoted as $X_{J_{i}+2:m_{i}:n_{i}},...,X_{i(m_{i}:m_{i}:n_{i})}$.

**Table 3:** Designed scenario of BAPCS schemes.

*k*	*T*	*n*	$N=(n_{1},n_{2},...,n_{k})$	$M=(m_{1},m_{2},...,n_{k})$
3	(1.2, 1.5, 0.9)	135	*N*_1_=(50, 40, 45)	*M*_1_=(45, 35, 40)
		195	*N*_2_=(70, 60, 65)	*M*_2_=(50, 40, 45)
		300	*N*_3_=(110, 90, 100)	*M*_3_=(70, 60, 65)
4	(1.2, 1.5, 0.9, 0.8)	190	*N*_4_=(50, 40, 45, 55)	*M*_4_=(45, 35, 40, 30)
		270	*N*_5_=(70, 60, 65, 75)	*M*_5_=(50, 40, 45, 55)
		420	*N*_6_=(110, 90, 100, 120)	*M*_6_=(70, 60, 65, 75)

We selected the true parameters of the model to be (α,β)=(1.5,0.5), and the values of the hyperparameters (η,ξ) are selected to be (0.01, 0.01). The choice of hyperparameter values is based on a weakly informative prior setting, which is performed by selecting small values for the shape and rate parameters, such as 0.01. This approach is commonly used to reflect unclear prior knowledge and to allow the data to control the posterior inference. The MCMC algorithm was implemented with 12,000 iterations,of which the first 2,000 iterations were discarded as burn-in. To reduce serial autocorrelation, every third sample was retained through a thinning procedure. The simulation results are given in Table 4–7. The main findings of the simulation study are summarized below:

**Table 4:** Point and interval estimates of parameter and reliability indices for CS-I and *k* = 3.

		MLE		MPSE		Bayes											
						SE		LINEX									
								*c* = 1		c=−1		${ACI}_{MLE}$		HPD		${ACI}_{MPSE}$	
(*N*,*M*)	Parameter	MSE	AB	MSE	AB	MSE	AB	MSE	AB	MSE	AB	AW	CP	AW	CP	AW	CP
$\left(N_{1},M_{1}\right)$	$\alpha_{1}$	0.0630	0.0549	0.0520	−0.0715	0.0546	0.0395	0.0491	0.0112	0.0625	0.0693	0.9434	0.962	0.9153	0.933	0.8677	0.928
	$\alpha_{2}$	0.1062	0.0920	0.0735	−0.0649	0.0893	0.0675	0.0769	0.0297	0.1069	0.1082	1.0921	0.940	1.0529	0.987	0.9826	0.920
	$\alpha_{3}$	0.0738	0.0538	0.0515	−0.0780	0.0678	0.0179	0.0615	−0.0129	0.0772	0.0506	0.9966	0.946	0.9514	0.933	0.9113	0.930
	β	0.0020	0.0144	0.0025	−0.0318	0.0024	0.0057	0.0024	0.0040	0.0024	0.0075	0.1586	0.950	0.2254	0.987	0.1484	0.862
	α	0.0247	0.0039	0.0320	−0.1172	0.0231	−0.0555	0.0239	−0.0679	0.0228	−0.0429	0.5898	0.946	0.6099	0.927	0.5428	0.874
	*S*(*x*)	0.0011	0.0063	0.0013	0.0190	0.0016	0.0198	0.0016	0.0191	0.0017	0.0205	0.1204	0.946	0.1470	0.933	0.1223	0.926
	*h*(*x*)	0.0682	0.0272	0.1012	−0.2249	0.0650	−0.0948	0.0690	−0.1299	0.0637	−0.0585	0.9835	0.950	1.0278	0.920	0.8938	0.834
	η					0.5537	0.2531	0.4676	0.1433	0.6699	0.3658						
	ξ					0.0447	0.2102	0.0360	0.1888	0.0582	0.2393						
$\left(N_{2},M_{2}\right)$	$\alpha_{1}$	0.0857	0.0901	0.0570	−0.0438	0.0538	0.0761	0.0469	0.0493	0.0627	0.1044	1.0436	0.962	0.8927	0.992	0.9391	0.926
	$\alpha_{2}$	0.1175	0.1224	0.0750	−0.0285	0.0697	0.0727	0.0602	0.0398	0.0828	0.1078	1.1848	0.964	0.9865	0.959	1.0550	0.938
	$\alpha_{3}$	0.0959	0.1134	0.0759	−0.0206	0.0763	0.0583	0.0679	0.0294	0.0876	0.0888	1.1143	0.960	0.9198	0.926	1.0058	0.928
	β	0.0024	0.0196	0.0021	−0.0157	0.0015	0.0102	0.0015	0.0095	0.0015	0.0109	0.1808	0.952	0.1418	0.950	0.1687	0.918
	α	0.0426	0.0487	0.0377	−0.0792	0.0236	−0.0147	0.0233	−0.0262	0.0243	−0.0031	0.7629	0.960	0.5853	0.934	0.6780	0.918
	*S*(*x*)	0.0011	−0.0008	0.0013	0.0162	0.0011	0.0114	0.0011	0.0109	0.0011	0.0119	0.1258	0.942	0.1235	0.942	0.1274	0.936
	*h*(*x*)	0.1341	0.1044	0.1167	−0.1396	0.0652	−0.0073	0.0625	−0.0376	0.0703	0.0242	1.3442	0.962	0.9512	0.934	1.1840	0.898
	η					0.9819	0.6095	0.7313	0.4629	1.2802	0.7540						
	ξ					0.0433	0.2068	0.0349	0.1857	0.0566	0.2359						
$\left(N_{3},M_{3}\right)$	$\alpha_{1}$	0.0665	0.0768	0.0426	−0.0460	0.0426	0.0384	0.0394	0.0202	0.0467	0.0573	0.9026	0.950	0.7342	0.917	0.8175	0.922
	$\alpha_{2}$	0.0711	0.0735	0.0464	−0.0396	0.0538	0.0437	0.0493	0.0227	0.0596	0.0656	0.9377	0.946	0.7880	0.909	0.8577	0.926
	$\alpha_{3}$	0.0583	0.0660	0.0456	−0.0442	0.0461	0.0657	0.0414	0.0455	0.0520	0.0867	0.9116	0.970	0.7747	0.959	0.8351	0.914
	β	0.0018	0.0125	0.0016	−0.0145	0.0009	0.0070	0.0009	0.0065	0.0009	0.0075	0.1499	0.924	0.1169	0.967	0.1421	0.916
	α	0.0304	0.0318	0.0272	−0.0769	0.0129	−0.0170	0.0129	−0.0246	0.0130	−0.0093	0.6482	0.942	0.4771	0.959	0.5852	0.882
	*S*(*x*)	0.0008	−0.0003	0.0010	0.0151	0.0006	0.0090	0.0006	0.0086	0.0006	0.0093	0.1078	0.950	0.1004	0.983	0.1089	0.914
	*h*(*x*)	0.0970	0.0678	0.0835	−0.1350	0.0353	−0.0150	0.0347	−0.0352	0.0367	0.0058	1.1406	0.942	0.7777	0.950	1.0233	0.886
	η					1.0802	0.6684	0.8195	0.5175	1.3951	0.8167						
	ξ					0.0432	0.2072	0.0349	0.1863	0.0560	0.2356						

**Table 5:** Point and interval estimates of parameter and reliability indices for CS-II and *k* = 3.

		MLE		MPSE		Bayes											
						SE		LINEX									
								*c* = 1		c=−1		${ACI}_{MLE}$		HPD		${ACI}_{MPSE}$	
(*N*,*M*)	Parameter	MSE	AB	MSE	AB	MSE	AB	MSE	AB	MSE	AB	AW	CP	AW	CP	AW	CP
$\left(N_{1},M_{1}\right)$	$\alpha_{1}$	0.0632	0.0368	0.0506	−0.0756	0.0585	−0.0096	0.0551	−0.0362	0.0641	0.0186	0.9327	0.948	0.8883	0.927	0.8655	0.922
	$\alpha_{2}$	0.0909	0.0630	0.0694	−0.0939	0.0884	0.0595	0.0762	0.0217	0.1058	0.1002	1.0736	0.944	1.0527	0.944	0.9633	0.906
	$\alpha_{3}$	0.1006	0.0597	0.0692	−0.0846	0.1255	−0.1031	0.1223	−0.1314	0.1317	−0.0728	1.0314	0.910	0.8975	0.866	0.9303	0.884
	β	0.0019	0.0100	0.0028	−0.0366	0.0020	−0.0029	0.0020	−0.0037	0.0020	−0.0021	0.1613	0.956	0.1536	0.903	0.1509	0.872
	α	0.0281	−0.0136	0.0389	−0.1289	0.0554	−0.1369	0.0575	−0.1487	0.0536	−0.1249	0.5967	0.918	0.5929	0.858	0.5689	0.846
	*S*(*x*)	0.0012	0.0091	0.0015	0.0208	0.0034	0.0386	0.0033	0.0379	0.0035	0.0393	0.1211	0.950	0.1414	0.898	0.1228	0.894
	*h*(*x*)	0.0800	−0.0043	0.1235	−0.2486	0.1413	−0.2038	0.1483	−0.2348	0.1367	−0.1717	1.0039	0.928	0.9557	0.866	0.9306	0.846
	η					0.7717	0.4552	0.6060	0.3159	0.9995	0.5935						
	ξ					0.0495	0.2207	0.0393	0.1969	0.0660	0.2541						
$\left(N_{2},M_{2}\right)$	$\alpha_{1}$	0.0605	0.0432	0.0464	−0.0734	0.0757	0.0932	0.0659	0.0651	0.0882	0.1229	0.8896	0.948	0.9102	0.959	0.8320	0.914
	$\alpha_{2}$	0.0817	0.0614	0.0566	−0.0938	0.0777	0.0779	0.0668	0.0430	0.0927	0.1152	1.0234	0.948	1.0107	0.959	0.9295	0.922
	$\alpha_{3}$	0.0909	0.0758	0.0707	−0.0628	0.0990	0.0367	0.0881	0.0034	0.1144	0.0727	1.0262	0.944	0.9812	0.876	0.9287	0.878
	β	0.0017	0.0103	0.0021	−0.0281	0.0018	0.0087	0.0018	0.0080	0.0018	0.0095	0.1494	0.938	0.1464	0.909	0.1427	0.868
	α	0.0277	0.0025	0.0340	−0.1208	0.0304	−0.0307	0.0301	−0.0440	0.0310	−0.0172	0.5957	0.934	0.6253	0.884	0.5742	0.869
	*S*(*x*)	0.0010	0.0055	0.0014	0.0212	0.0013	0.0155	0.0013	0.0149	0.0014	0.0160	0.1147	0.924	0.1276	0.917	0.1174	0.910
	*h*(*x*)	0.0795	0.0203	0.1036	−0.2220	0.0869	−0.0310	0.0839	−0.0673	0.0935	0.0070	1.0100	0.932	1.0364	0.909	0.9916	0.872
	η					0.9831	0.6259	0.7180	0.4712	1.3525	0.7846						
	ξ					0.0440	0.2083	0.0353	0.1868	0.0576	0.2378						
$\left(N_{3},M_{3}\right)$	$\alpha_{1}$	0.0405	0.0411	0.0335	−0.0535	0.0418	0.0461	0.0383	0.0277	0.0462	0.0652	0.7482	0.948	0.7365	0.975	0.7021	0.932
	$\alpha_{2}$	0.0490	0.0569	0.0382	−0.0724	0.0396	0.0235	0.0366	0.0020	0.0439	0.0460	0.8325	0.962	0.7980	0.950	0.7609	0.928
	$\alpha_{3}$	0.0523	0.0411	0.0467	−0.0873	0.0757	−0.0403	0.0723	−0.0610	0.0807	−0.0185	0.8367	0.944	0.7726	0.864	0.7607	0.890
	β	0.0011	0.0067	0.0016	−0.0252	0.0011	0.0004	0.0011	−0.0001	0.0011	0.0009	0.1225	0.938	0.1185	0.926	0.1164	0.888
	α	0.0187	0.0105	0.0228	−0.1010	0.0233	−0.0550	0.0236	−0.0634	0.0230	−0.0465	0.4984	0.936	0.4976	0.917	0.4687	0.850
	*S*(*x*)	0.0006	0.0016	0.0008	0.0165	0.0011	0.0165	0.0010	0.0161	0.0011	0.0169	0.0948	0.936	0.1028	0.901	0.0970	0.920
	*h*(*x*)	0.0552	0.0262	0.0727	−0.1887	0.0634	−0.0794	0.0645	−0.1024	0.0636	−0.0556	0.8450	0.936	0.8280	0.909	0.7891	0.864
	η					1.0153	0.5757	0.7588	0.4208	1.3624	0.7330						
	ξ					0.0459	0.2130	0.0368	0.1909	0.0601	0.2435						

**Table 6:** Point and interval estimates of parameter and reliability indices for CS-I and *k* = 4.

		MLE		MPSE		Bayes											
						SE		LINEX									
								*c* = 1		c=−1		${ACI}_{MLE}$		HPD		${ACI}_{MPSE}$	
(*N*,*M*)	Parameter	MSE	AB	MSE	AB	MSE	AB	MSE	AB	MSE	AB	AW	CP	AW	CP	AW	CP
$\left(N_{4},M_{4}\right)$	$\alpha_{1}$	0.0730	0.0685	0.0508	−0.0579	0.0808	0.0763	0.0710	0.0469	0.0936	0.1076	0.9811	0.963	0.9323	0.952	0.8952	0.922
	$\alpha_{2}$	0.0877	0.0769	0.0651	−0.0616	0.0801	0.0753	0.0683	0.0384	0.0968	0.1150	1.1101	0.957	1.0444	0.960	1.0056	0.914
	$\alpha_{3}$	0.0999	0.0596	0.0757	−0.0661	0.0562	0.0598	0.0490	0.0280	0.0665	0.0935	1.0313	0.904	0.9715	0.944	0.9515	0.912
	$\alpha_{4}$	0.1398	0.0892	0.0962	−0.0669	0.1060	0.1136	0.0868	0.0684	0.1331	0.1629	1.3102	0.958	1.1535	0.952	1.1648	0.926
	β	0.0021	0.0134	0.0024	−0.0288	0.0018	0.0145	0.0018	0.0139	0.0018	0.0152	0.1606	0.932	0.1360	0.887	0.1481	0.868
	α	0.0300	−0.0042	0.0383	−0.1240	0.0212	−0.0318	0.0213	−0.0425	0.0213	−0.0210	0.6085	0.911	0.5655	0.911	0.5488	0.872
	*S*(*x*)	0.0011	0.0082	0.0015	0.0222	0.0012	0.0163	0.0012	0.0158	0.0012	0.0168	0.1120	0.905	0.1220	0.927	0.1139	0.893
	*h*(*x*)	0.0905	0.0161	0.1191	−0.2270	0.0577	−0.0254	0.0565	−0.0535	0.0607	0.0036	1.0608	0.912	0.9160	0.952	0.9434	0.874
	η					1.0637	0.6518	0.7963	0.5011	1.3870	0.8008						
	ξ					0.0232	0.1512	0.0196	0.1392	0.0281	0.1663						
$\left(N_{5},M_{5}\right)$	$\alpha_{1}$	0.0695	0.0568	0.0538	−0.0606	0.0591	0.0617	0.0530	0.0362	0.0671	0.0883	0.9742	0.957	0.8698	0.968	0.8903	0.935
	$\alpha_{2}$	0.0831	0.0613	0.0628	−0.0541	0.0993	0.0981	0.0857	0.0643	0.1174	0.1341	1.0849	0.964	0.9953	0.927	0.9942	0.927
	$\alpha_{3}$	0.0871	0.0867	0.0623	−0.0487	0.0702	0.0670	0.0620	0.0384	0.0811	0.0973	1.0465	0.952	0.9206	0.935	0.9448	0.939
	$\alpha_{4}$	0.0660	0.0651	0.0530	−0.0371	0.0521	0.0873	0.0456	0.0631	0.0603	0.1126	0.9379	0.953	0.8489	0.944	0.8677	0.943
	β	0.0016	0.0106	0.0018	−0.0218	0.0008	0.0070	0.0008	0.0065	0.0008	0.0075	0.1494	0.951	0.1177	0.976	0.1401	0.911
	α	0.0245	0.0073	0.0290	−0.0975	0.0174	−0.0195	0.0174	−0.0278	0.0176	−0.0111	0.6114	0.950	0.4992	0.952	0.5543	0.922
	*S*(*x*)	0.0008	0.0041	0.0010	0.0175	0.0008	0.0102	0.0008	0.0098	0.0008	0.0106	0.1057	0.937	0.1062	0.944	0.1068	0.916
	*h*(*x*)	0.0758	0.0287	0.0921	−0.1768	0.0446	−0.0181	0.0439	−0.0399	0.0465	0.0041	1.0760	0.955	0.8087	0.960	0.9666	0.893
	η					1.1745	0.7055	0.8584	0.5513	1.5860	0.8645						
	ξ					0.0227	0.1500	0.0193	0.1383	0.0273	0.1645						
$\left(N_{6},M_{6}\right)$	$\alpha_{1}$	0.0520	0.0490	0.0397	−0.0395	0.0378	0.0577	0.0343	0.0395	0.0421	0.0765	0.8424	0.957	0.7379	0.952	0.7864	0.958
	$\alpha_{2}$	0.0570	0.0459	0.0458	−0.0378	0.0431	0.0631	0.0387	0.0419	0.0489	0.0853	0.8819	0.956	0.7963	0.960	0.8277	0.947
	$\alpha_{3}$	0.0560	0.0540	0.0423	−0.0414	0.0559	0.0636	0.0510	0.0438	0.0619	0.0840	0.8653	0.947	0.7645	0.935	0.8040	0.943
	$\alpha_{4}$	0.0507	0.0531	0.0383	−0.0411	0.0469	0.0878	0.0421	0.0700	0.0526	0.1062	0.8273	0.949	0.7292	0.935	0.7685	0.934
	β	0.0012	0.0076	0.0012	−0.0161	0.0008	0.0092	0.0008	0.0089	0.0008	0.0095	0.1264	0.934	0.1001	0.895	0.1206	0.913
	α	0.0206	0.0093	0.0211	−0.0746	0.0113	−0.0016	0.0111	−0.0057	0.0115	0.0057	0.5425	0.944	0.4131	0.960	0.5013	0.921
	*S*(*x*)	0.0006	0.0023	0.0007	0.0134	0.0005	0.0053	0.0005	0.0051	0.0005	0.0056	0.0924	0.935	0.0856	0.927	0.0931	0.934
	*h*(*x*)	0.0650	0.0271	0.0664	−0.1344	0.0296	0.0134	0.0284	−0.0018	0.0314	0.0289	0.9542	0.945	0.6743	0.960	0.8763	0.943
	η					1.0007	0.6745	0.7591	0.5287	1.3062	0.8225						
	ξ					0.0229	0.1508	0.0194	0.1389	0.0276	0.1656						

**Table 7:** Point and interval estimates of parameter and reliability indices for CS-III and *k* = 4.

		MLE		MPSE		Bayes											
						SE		LINEX									
								*c* = 1		c=−1		${ACI}_{MLE}$		HPD		${ACI}_{MPSE}$	
(*N*,*M*)	Parameter	MSE	AB	MSE	AB	MSE	AB	MSE	AB	MSE	AB	AW	CP	AW	CP	AW	CP
$\left(N_{4},M_{4}\right)$	$\alpha_{1}$	0.0662	0.0441	0.0510	−0.0714	0.0620	0.0276	0.0561	−0.0001	0.0702	0.0567	0.9308	0.945	0.8986	0.911	0.8613	0.911
	$\alpha_{2}$	0.0894	0.0650	0.0604	−0.0857	0.0687	0.0376	0.0611	0.0023	0.0801	0.0753	1.0672	0.950	1.0232	0.960	0.9620	0.924
	$\alpha_{3}$	0.0944	0.0521	0.0667	−0.0882	0.1147	−0.0457	0.1088	−0.0751	0.1238	−0.0142	1.0112	0.912	0.9184	0.815	0.9151	0.881
	$\alpha_{4}$	0.1041	0.0643	0.0693	−0.1029	0.0841	0.0612	0.0716	0.0187	0.1031	0.1073	1.1495	0.955	1.1199	0.968	1.0257	0.914
	β	0.0016	0.0118	0.0025	−0.0379	0.0012	0.0000	0.0012	−0.0006	0.0012	0.0007	0.142	0.935	0.1350	0.919	0.1313	0.854
	α	0.0232	−0.0203	0.0340	−0.1391	0.0404	−0.1027	0.0417	−0.1129	0.0393	−0.0924	0.5288	0.892	0.5490	0.782	0.5150	0.823
	*S*(*x*)	0.0011	0.0107	0.0013	0.0221	0.0024	0.0297	0.0024	0.0292	0.0024	0.0303	0.1087	0.913	0.1265	0.839	0.1103	0.901
	*h*(*x*)	0.0642	−0.0107	0.1107	−0.2657	0.0988	−0.1498	0.1031	−0.1763	0.0962	−0.1225	0.8870	0.904	0.8869	0.766	0.8706	0.861
	η					0.9761	0.5508	0.7587	0.4204	1.2610	0.6830						
	ξ					0.0253	0.1580	0.0213	0.1450	0.0309	0.1744						
$\left(N_{5},M_{5}\right)$	$\alpha_{1}$	0.0515	0.0373	0.0447	−0.0728	0.0788	0.0674	0.0704	0.0412	0.0895	0.0949	0.8752	0.955	0.8785	0.927	0.8130	0.918
	$\alpha_{2}$	0.0798	0.0553	0.0562	−0.0821	0.1014	0.0989	0.0872	0.0641	0.1202	0.1361	1.0030	0.947	1.0080	0.895	0.9135	0.911
	$\alpha_{3}$	0.0739	0.0694	0.0578	−0.0783	0.0905	0.0414	0.0810	0.0099	0.1037	0.0749	0.9906	0.943	0.9564	0.879	0.8920	0.910
	$\alpha_{4}$	0.0518	0.0463	0.0440	−0.0591	0.0555	0.0685	0.0495	0.0449	0.0633	0.0932	0.8418	0.954	0.8377	0.935	0.7846	0.918
	β	0.0011	0.0098	0.0019	−0.0308	0.0014	0.0133	0.0014	0.0127	0.0014	0.0138	0.1249	0.949	0.1243	0.919	0.1212	0.871
	α	0.0163	−0.0038	0.0274	−0.1153	0.0234	−0.0367	0.0234	−0.0457	0.0235	−0.0276	0.4869	0.934	0.5163	0.903	0.4704	0.898
	*S*(*x*)	0.0007	0.0057	0.0010	0.0184	0.0012	0.0167	0.0011	0.0163	0.0012	0.0171	0.0961	0.937	0.1078	0.855	0.0979	0.901
	*h*(*x*)	0.0460	0.0102	0.0880	−0.2192	0.0620	−0.0328	0.0608	−0.0572	0.0648	−0.0077	0.8218	0.933	0.8517	0.927	0.8106	0.877
	η					1.1163	0.6759	0.8578	0.5312	1.4488	0.8218						
	ξ					0.0238	0.1535	0.0201	0.1412	0.0289	0.1689						
$\left(N_{6},M_{6}\right)$	$\alpha_{1}$	0.0364	0.0369	0.0309	−0.0521	0.0488	0.0684	0.0442	0.0499	0.0544	0.0877	0.7383	0.956	0.7434	0.935	0.6956	0.936
	$\alpha_{2}$	0.0446	0.0431	0.0350	−0.0681	0.0645	0.0592	0.0585	0.0371	0.0721	0.0822	0.8116	0.963	0.8054	0.911	0.7524	0.933
	$\alpha_{3}$	0.0531	0.0456	0.0438	−0.0838	0.0503	−0.0043	0.0480	−0.0243	0.0538	0.0165	0.8154	0.943	0.7662	0.887	0.7425	0.881
	$\alpha_{4}$	0.0363	0.0327	0.0309	−0.0624	0.0310	0.0332	0.0288	0.0168	0.0338	0.0501	0.7123	0.950	0.7021	0.944	0.6681	0.928
	β	0.0007	0.0056	0.0013	−0.0254	0.0007	0.0063	0.0007	0.0060	0.0008	0.0067	0.1030	0.950	0.1004	0.968	0.0982	0.921
	α	0.0106	0.0003	0.0184	−0.0968	0.0164	−0.0287	0.0165	−0.0346	0.0164	−0.0226	0.4095	0.958	0.4215	0.871	0.3923	0.895
	*S*(*x*)	0.0004	0.0028	0.0006	0.0150	0.0007	0.0113	0.0007	0.0111	0.0007	0.0116	0.0802	0.948	0.0863	0.903	0.0817	0.917
	*h*(*x*)	0.0304	0.0095	0.0597	−0.1832	0.0452	−0.0309	0.0449	−0.0473	0.0460	−0.0142	0.6906	0.958	0.6974	0.879	0.6783	0.894
	η					1.1563	0.7153	0.8981	0.5882	1.4722	0.8438						
	ξ					0.0239	0.1540	0.0202	0.1418	0.0290	0.1693

- The simulation study demonstrates that all estimation approaches perform well in terms of MSE and AB, with both metrics decreasing as the effective sample size increases.
- Although the differences between methods may be numerically small, such variations can still offer meaningful guidance on the relative efficiency and robustness of the estimation approaches.
- The hierarchical Bayesian model consistently outperforms both MLE and MPSE, achieving lower MSE and AB across all censoring schemes and sample sizes. This superiority is particularly evident under the LINEX loss function with *c* = 1, which yields more stable and accurate estimates.
- MPSE demonstrates competitive or improved performance over MLE in terms of lower MSE and AB, especially for parameters $\alpha_{i}$.
- Additionally, Bayesian estimates for *S*(*x*) and *h*(*x*) exhibit significantly lower bias and MSE compared to MLE and MPSE, highlighting the advantage of Bayesian methods in modeling reliability functions.
- Regarding interval estimation, HPD intervals consistently achieve higher CPs and narrower AWs than ${\text{ACI}}_{MLE}$ and ${\text{ACI}}_{MPSE}$.
- The study also highlights the impact of censoring schemes on estimation precision, with Bayesian estimators maintaining more stable performance across different censoring scenarios, whereas MLE and MPSE estimates show greater variability.
- Furthermore, the hierarchical Bayesian model provides robust estimates for η and ξ, with low AB and MSE across all test cases. The estimated DDF, given by $\hat{\eta }/{\hat{\xi }}^{2}$, plays a critical role in reliability inference, where higher values indicate greater variability in $\alpha_{i}$ across test facilities, leading to reduced estimation accuracy. Conversely, a lower DDF improves the precision of estimating $\alpha_{i}$.
- Larger group sizes (*k* = 4) tend to improve the accuracy and precision of the estimates, as seen in the lower MSE and AB values compared to smaller groups (*k* = 3). This suggests that incorporating additional test facilities enhances the robustness of parameter estimation.

Overall, these findings strongly support the use of hierarchical Bayesian models for reliability estimation under BAPCS, particularly when dealing with censored data or variations across multiple testing environments. The superior accuracy, improved interval estimation, and stability across censoring schemes make Bayesian methods the preferred approach for practical reliability studies.

## 7. Application to real-world data

While the proposed methodology is motivated by highly reliable systems, it remains applicable to real-world data with moderate variability. Such variability is common in practice due to heterogeneous testing conditions. We now demonstrate the utility of the method using two real data sets.

### 7.1 Cancer patients data

The first dataset examines the survival times of the patients, where some receive supplemental ascorbate alongside their treatment, while others do not. Provides average survival durations for different types of cancer, including breast, kidney, and ovary. Data are recorded from the initial hospital visit for cancer treatment, typically in an advanced stage. Previous studies, such as [32] and [33], have explored applications of this data set in various contexts. The initial data are presented in Table 8.

**Table 8:** Complete cancer patients data.

Kidney	2, 5, 6, 8, 8, 8, 14, 16, 17, 26, 27, 29, 29, 31, 41, 49, 55, 60, 65, 68,
	69, 76, 81, 82, 83, 95, 106, 114, 117, 125
Ovary	1, 2, 10, 15, 18, 19, 21, 21, 22, 22, 32, 33, 36, 38, 39, 40, 48, 49, 53, 68,
	85, 86, 97, 99, 106, 107, 160
Breast	1, 2, 2, 3, 4, 6, 6, 7, 10, 12, 12, 14, 14, 15, 15, 15, 16, 16, 17, 19,
	19, 22, 26, 28, 29, 33, 34, 38, 41, 45, 45, 48, 50, 52, 55, 61, 65, 69, 71, 76,
	81, 94, 101, 102, 107, 109, 131, 183, 190, 251

To assess the suitability of the Chen distribution in modeling cancer patient data, we performed a goodness-of-fit analysis. In addition to the Chen model, commonly used lifetime distributions were fitted to the data, including the Burr XII, Weibull, Gompertz, log-logistic (LL), and lognormal (LN) distributions. The results, summarized in Table 9, report the Kolmogorov–Smirnov (KS) statistic and its associated p-value, together with the Akaike Information Criterion (AIC) and Bayesian Information Criterion (BIC). Across all three cancer datasets, the Chen distribution shows consistently competitive performance. Although some of the competing model occasionally yield slightly smaller AIC or BIC, the p-values of KS statistic for the Chen distribution remain consistently higher than 0.8293. This indicates a fully compatible performance of the Chen distribution with the empirical behavior of each block. To further examine the adequacy of the Chen distribution, Fig 3 displays the histograms with estimated PDFs and the empirical vs fitted survival functions, while Fig 4 presents the associated QQ and PP plots. These graphical diagnostics support the goodness-of-fit test results and confirm that Chen distribution provides an appropriate and reliable fit to the cancer patients’ data.

**Table 9:** Goodness-fit-test for cancer patients’ data.

Data	Model	$\hat{\alpha }$	$\hat{\beta }$	KS statistic	p-value	AIC	BIC
Kidney	Chen	0.0180	0.3420	0.1108	0.8545	296.656	299.459
	Burr XII	0.0127	22.5723	0.3488	0.0014	348.471	351.273
	Weibull	1.2395	0.0072	0.1174	0.8024	297.352	300.154
	Gompertz	0.0120	0.9752	0.1124	0.8427	294.979	297.781
	LL	1.5951	36.6965	0.1240	0.7455	304.373	307.176
	LN	3.4910	1.0817	0.1501	0.5086	303.310	306.113
Ovary	Chen	0.0219	0.3322	0.1203	0.8293	267.338	269.929
	Burr XII	0.0024	119.50	0.4303	0.0001	314.177	316.769
	Weibull	1.2315	0.0076	0.0961	0.9644	266.648	269.240
	Gompertz	0.0075	1.9651	0.0980	0.9576	266.316	268.907
	LL	1.7340	36.4848	0.0970	0.9612	271.279	273.870
	LN	3.4732	1.1235	0.1538	0.5453	274.466	277.058
Breast	Chen	0.0450	0.2872	0.0819	0.8905	500.541	504.365
	Burr XII	0.0035	85.8648	0.3498	0.0000	553.825	557.649
	Weibull	0.9590	0.0243	0.0844	0.8678	493.520	497.344
	Gompertz	0.0002	115.092	0.1018	0.6775	493.732	497.556
	LL	1.4184	28.9563	0.0714	0.9606	497.197	501.021
	LN	3.2926	1.2362	0.0812	0.8963	496.366	500.190

The dataset, presented in Table 8, is categorized into three blocks, each representing a distinct cancer type (*k* = 3). The original sample sizes for these blocks are $\left(n_{1},n_{2},n_{3})=(50,30,27\right)$. From this dataset, censored samples are generated using the BAPCS, resulting in effective sample sizes of $\left(m_{1},m_{2},m_{3})=(30,20,15\right)$ with experimental duration times *T* = (80, 60, 40). Table 10 presents these generated samples under different censoring schemes. If an observation extended beyond the predetermined experimental time $T_{i}$ (*i* = 1,2,3), the censoring scheme was adjusted accordingly, as detailed in the same table. To account for variability across different test facilities, the BAPCS cancer dataset is analyzed by applying the Chen distribution model to each block individually. The model incorporates facility-specific scale parameters $\alpha_{i}$ (*i* = 1,2,3), alongside a common shape parameter β. Table 11 presents a comparative summary of the parameter estimates obtained through MLE, Bayesian inference, and MPSE, as well as the corresponding reliability measures: the survival function *S*(80) and hazard function *h*(80).

**Table 10:** APCS samples from cancer patients’ data.

Data	$\left(n_{i},m_{i}\right)$	Scheme	$\left(T_{i},J_{i}\right)$	Adjusted scheme	APCS sample									
Breast	(50, 30)	$\left(0^{*15},2^{*5},1^{*10}\right)$	(80, 25)	$\left(0^{*15},2^{*5},1^{*5},0^{*4},5\right)$	1	2	2	3	4	6	6	7	10	12
					12	14	14	15	15	15	17	22	29	38
					45	50	55	65	71	81	94	101	102	107
Kidney	(30, 20)	$\left(0^{*15},2^{*5}\right)$	(60, 16)	$\left(0^{*15},2,0^{*3},8\right)$	2	5	6	8	8	8	14	16	17	26
					27	29	29	31	41	49	65	68	69	76
Ovary	(27, 15)	$\left(0^{*3},1^{*12}\right)$	(40, 10)	$\left(0^{*3},1^{*7},0^{*4},5\right)$	1	2	10	15	19	21	22	33	38	40
					49	53	68	85	86					

**Table 11:** Point and interval estimates for cancer patients data.

Parameters	MLE	MPSE	Bayes Estimates					
			SE	Lindley				
				*c* = 1	c=−1	${ACI}_{MLE}$	HPD	${ACI}_{MPSE}$
$\alpha_{1}$	0.0248	0.0298	0.0245	0.0245	0.0246	(0.0092, 0.0404)	(0.0123,0.0423)	(0.0121, 0.0474)
$\alpha_{2}$	0.0275	0.0325	0.0230	0.0230	0.0231	(0.0096, 0.0454)	(0.0112,0.0409)	(0.0122, 0.0527)
$\alpha_{3}$	0.0229	0.0269	0.0205	0.0205	0.0205	(0.0068, 0.0390)	(0.0093,0.0379)	(0.0088, 0.0451)
α	0.0249	0.0295	0.0215	0.0215	0.0215	(0.0110, 0.0387)	(0.0116,0.0355)	(0.0142, 0.0449)
β	0.3069	0.2930	0.2935	0.2933	0.2936	(0.2736, 0.3401)	(0.2606,0.3251)	(0.2596, 0.3265)
*S*(*x*)	0.3231	0.3452	0.4687	0.4674	0.4701	(0.2282, 0.4181)	(0.3711,0.5764)	(0.2480, 0.4424)
*h*(*x*)	0.0170	0.0145	0.0105	0.0105	0.0105	(0.0102, 0.0238)	(0.0067,0.0152)	(0.0084, 0.0205)
η			0.0952	0.0916	0.0991		(0.0038,0.3229)	
ξ			15.654	2.7327	18.148		(0.3284,61.206)	

To assess the existence and uniqueness of the MLEs, we plot the profile log-likelihood functions for each parameter, in Fig 5. The resulting curves exhibit smooth, concave shapes with well-defined peaks, confirming that the likelihood function attains a unique maximum for each parameter. Bayesian estimates are derived using the MCMC technique, implemented over 55,000 iterations. To ensure convergence and reduce autocorrelation, the first 5,000 iterations are discarded as burn-in, and a thinning interval of 5 is applied. The analysis employs weakly informative priors with hyperparameters η and ξ both set to 0.1.

To assess the convergence of the MCMC chains, two diagnostic plots are examined. The marginal posterior density estimates exhibit a smooth and well-distributed shape, indicating sufficient exploration of the parameter space, while the trace plots of the parameters confirm that the chains fluctuate consistently within the same region without discernible trends, suggesting proper mixing and stable convergence. These results highlight the practical applicability and robustness of the proposed estimation methods for modeling cancer patient data, offering reliable insights into system reliability and failure behavior across heterogeneous testing environments. The corresponding diagnostic plots are provided in S1–S2 Figs.

Fig 6 further confirms the robustness of the Bayesian analysis. Even with extremely diffuse priors such as Gamma(0.001,0.001), the posterior mass is almost indistinguishable from that obtained under more informative priors. This behavior indicates that the data-rather than the priors-dominate the posterior inference. From Table 12, across all priors, β posteriors nearly coincide (CV ≈ 0.003–0.004; relative range ≈ 0.01–0.014), indicating β is effectively insensitive to prior specification. The $\alpha_{i}$ posteriors for cancer datasets show low sensitivity (CV typically 0.016–0.020; relative range < 0.07), with tight overlap of density curves. Hyperparameter η exhibit only modest variation (CV ≈ 0.026; the posterior mass shifts are small and do not affect the main inferences. β trace plot in Fig 7 show stable mixing and convergence to the same region under all priors, reinforcing robustness. These results demonstrate that the posterior inferences for β and $\alpha_{i}$ are dominated by the data rather than the priors; the inferential conclusions reported in this work are stable to wide prior perturbations.

**Table 12:** Sensitivity metrics for model parameters for Cancer patients data.

Parameter	CV of Means	Relative Range
β	0.003817	0.013597
$\alpha_{1}$	0.020104	0.069705
$\alpha_{2}$	0.019270	0.065730
$\alpha_{3}$	0.016287	0.056942
η	0.026290	0.071300

### 7.2 Time to breakdown data

The second data extracted from [34], consists of times to oil breakdown under high test voltages. The experimental setup involved two parallel plate electrodes with a fixed area and a gap, with the electrical stress represented by constant voltage levels because of the consistent geometry of the electrodes. The data set includes breakdown times recorded at four different stress levels: 30, 32, 34, and 36 kilovolts. Data are presented in Table 13.

**Table 13:** Time to Breakdown data.

30 kv	7.74, 17.05, 20.46, 21.02, 22.66, 43.4, 47.30, 139.07, 144.12, 175.88, 194.90
32 kv	0.27, 0.4, 0.69, 0.79, 2.75, 3.91, 9.88, 13, 95, 15.93, 27.8, 53.24, 82.85, 89.29, 100.58, 215.10
34 kv	0.19, 0.78, 0.96, 1.31, 2.78, 3.16, 4.15, 4.67, 4.85, 6.50, 7.35, 8.01, 8.27, 12.06, 31.75, 32.52, 33.91, 36.71, 72.89
36 kv	0.35, 0.59, 0.96, 0.99, 1.69, 1.97, 2.07, 2.58, 2.71, 2.90, 3.67, 3.99, 5.35, 13.77, 25.50

To examine the performance of the Chen distribution in modeling the time to breakdown data, a goodness-of-fit analysis has been conducted for the four voltage levels (30, 32, 34 and 36 kV) and presented in Table 14. Across all blocks, the Chen distribution provides a satisfactory and competitive fit, with high KS p-values indicating close agreement between the empirical data and the fitted model. Although some alternative models achieve slightly smaller AIC and BIC values in isolated cases, the overall performance of the Chen distribution remains fully adequate for representing the data. The graphical assessments in Figs 8 and 9 further support this conclusion.

**Table 14:** Goodness-fit-test for time to Breakdown data.

Data	Model	$\hat{\alpha }$	$\hat{\beta }$	KS statistic	p-value	AIC	BIC
30 kv	Chen	0.0251	0.2941	0.2208	0.6568	122.299	123.095
	Burr XII	0.0309	8.4688	0.4330	0.0324	139.581	140.377
	Weibull	1.0588	0.0100	0.2166	0.6802	121.157	121.953
	Gompertz	0.0024	4.5460	0.2273	0.6207	121.027	121.823
	LL	1.5333	44.6332	0.2146	0.6913	121.708	122.504
	LN	3.8220	1.0595	0.2169	0.6788	120.572	121.368
32 kv	Chen	0.1020	0.2400	0.1336	0.9377	148.736	150.281
	Burr XII	0.3162	1.1886	0.2106	0.4771	154.232	155.777
	Weibull	0.5775	0.1413	0.1491	0.8692	148.223	149.768
	Gompertz	0.0002	121.35	0.2917	0.1314	157.521	159.066
	LL	0.7723	12.1728	0.1421	0.9033	150.901	152.446
	LN	2.3694	2.1313	0.1493	0.8677	149.444	150.989
34 kv	Chen	0.1212	0.3013	0.1904	0.4960	143.708	145.597
	Burr XII	0.2936	1.7379	0.2226	0.3032	146.897	148.786
	Weibull	0.7708	0.1452	0.1613	0.7059	140.772	142.661
	Gompertz	0.0001	478.773	0.2475	0.1949	143.272	145.16
	LL	1.1735	6.2537	0.1338	0.8858	141.333	143.222
	LN	1.7864	1.4845	0.1331	0.8896	140.816	142.705
36 kv	Chen	0.2088	0.3595	0.2179	0.4747	84.177	85.593
	Burr XII	0.3766	2.4590	0.1720	0.7665	76.474	77.890
	Weibull	0.8891	0.2738	0.1917	0.6397	79.383	80.799
	Gompertz	0.0005	455.49	0.2215	0.4536	79.852	81.268
	LL	1.6765	2.3750	0.0953	0.9992	75.678	77.094
	LN	0.9022	1.0723	0.1267	0.9696	75.729	77.145

The dataset, presented in Table 13, is categorized into four blocks, each corresponding to a distinct voltage level (*k* = 4). The original sample sizes for these blocks are given as $\left(n_{1},n_{2},n_{3},n_{4})=(11,16,19,15\right)$. From this dataset, censored samples are generated using the BAPCS, resulting in effective sample sizes of $\left(m_{1},m_{2},m_{3},m_{4})=(7,10,13,9\right)$ with experimental duration times *T* = (40,30,12,3). Table 15 presents these generated samples under different censoring schemes. If an observation extended beyond the predetermined experimental time $T_{i}$ (*i* = 1,2,3,4), the censoring scheme was adjusted accordingly, as detailed in the same table.

**Table 15:** APCS samples from time to breakdown data.

Data	$\left(n_{i},m_{i}\right)$	Scheme	$\left(T_{i},J_{i}\right)$	Adjusted scheme	APCS sample								
30 kv	(11, 7)	$\left(0^{*3},1^{*4}\right)$	(40, 4)	$\left(0^{*3},1,0^{*2},3\right)$	7.74	17.05	20.46	21.02	43.4	47.3	139.07		
32 kv	(16, 10)	$\left(0^{*4},1^{*6}\right)$	(30, 7)	$\left(0^{*4},1^{*3},0^{*2},3\right)$	0.27	0.4	0.69	0.79	2.75	9.88	15.93	53.24	82.85
					89.29								
34 kv	(19, 13)	$\left(0^{*7},1^{*6}\right)$	(12, 10)	$\left(0^{*7},1^{*3},0^{*2},3\right)$	0.19	0.78	0.96	1.31	2.78	3.16	4.15	4.67	6.5
					8.01	12.06	31.75	32.52					
36 kv	(15, 9)	$\left(0^{*4},1^{*4},2\right)$	(3, 7)	$\left(0^{*4},1^{*3},0,3\right)$	0.35	0.59	0.96	0.99	1.69	2.07	2.71	3.67	3.99

**Fig 10:**
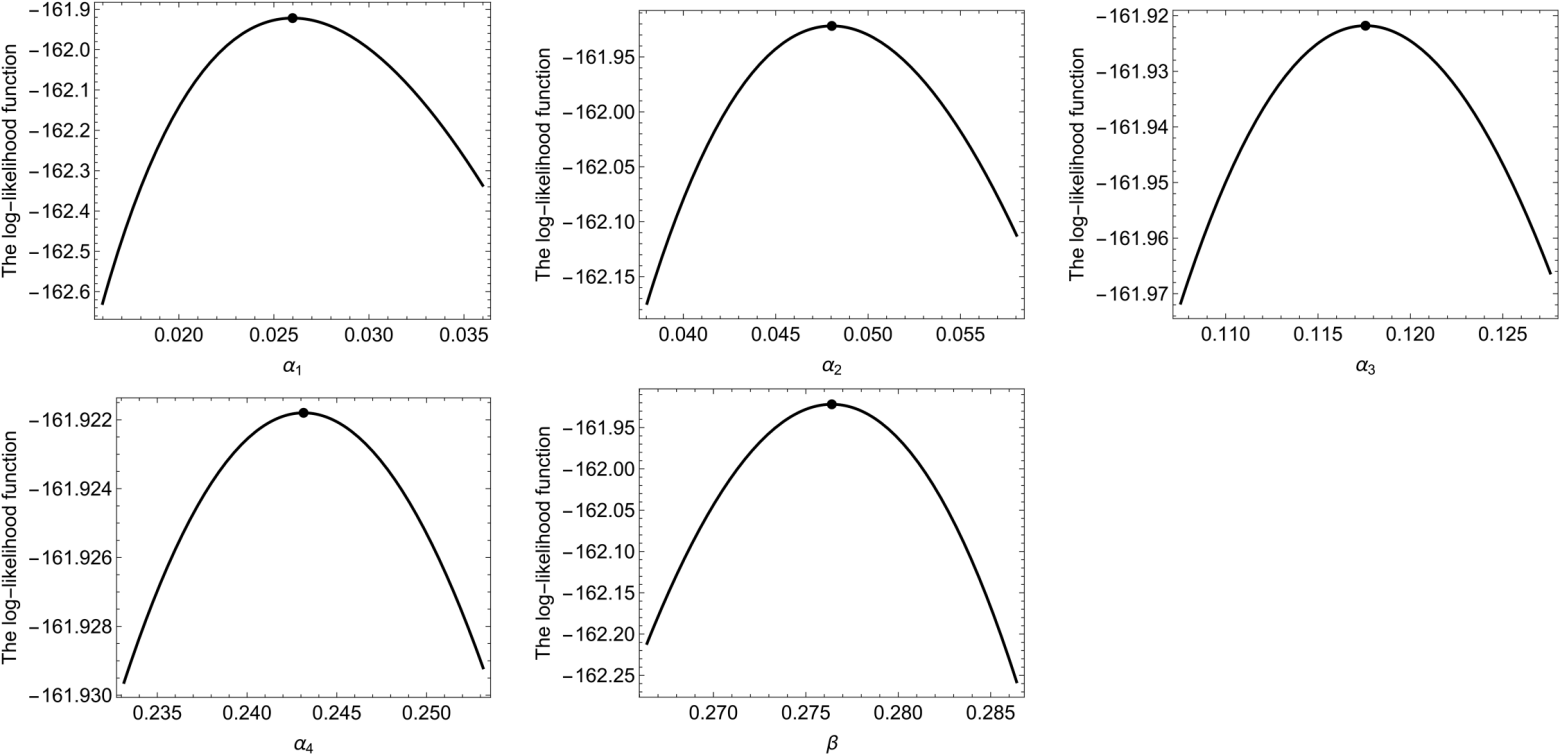
The profile of the log-likelihood function for all parameters for time to breakdown data.

For each block, considering the differences in experimental conditions across test facilities, the time to breakdown data under the BAPCS is analyzed using the Chen model with parameters $\alpha_{i}$ (*i* = 1,2,3,4), along with the common shape parameter β. Table 16 summarizes the MLEs, Bayesian estimates, and MPSEs of the parameters, in addition to reliability indices *S*(20) and *h*(20). To ensure that the MLEs of the parameters exist and are unique, the profile log-likelihood function for each parameter is plotted in Fig 10, which shows clear peaks and smooth curves. The Bayesian estimates are obtained using the MCMC method, executed over 55,000 iterations. The initial 5,000 iterations are discarded as burn-in, and a thinning interval of 5 is applied to mitigate autocorrelation. Weakly informative priors are used, with hyperparameters η and ξ both set to 0.1.

**Table 16:** Point and interval estimates for time to breakdown data.

Parameters	MLE	MPSE	Bayes Estimates					
			SE	Lindley				
				*c* = 1	c=−1	${ACI}_{MLE}$	HPD	${ACI}_{MPSE}$
$\alpha_{1}$	0.0260	0.0376	0.0229	0.0228	0.0229	(0.0000,0.0542)	(0.0061, 0.0547)	(0.0010, 0.0742)
$\alpha_{2}$	0.0480	0.0647	0.0424	0.0422	0.0426	(0.0052, 0.0909)	(0.0150, 0.0893)	(0.0124, 0.1169)
$\alpha_{3}$	0.1176	0.1353	0.0971	0.0966	0.0976	(0.0428, 0.1924)	(0.0466, 0.1719)	(0.0525, 0.2180)
$\alpha_{4}$	0.2431	0.2440	0.1869	0.1849	0.1890	(0.0832, 0.4031)	(0.0863, 0.3293)	(0.0837, 0.4043)
α	0.0443	0.0624	0.0321	0.0320	0.0323	(0.0147, 0.0738)	(0.0084, 0.0715)	(0.0270, 0.0978)
β	0.2764	0.2478	0.2615	0.2612	0.2618	(0.2292, 0.3236)	(0.2134, 0.3078)	(0.2006, 0.2950)
*S*(*x*)	0.6755	0.6390	0.7876	0.7849	0.7902	(0.5666, 0.7845)	(0.6380, 0.9182)	(0.5343, 0.7437)
*h*(*x*)	0.0138	0.0133	0.0077	0.0077	0.0077	(0.0088, 0.0188)	(0.0030, 0.0130)	(0.0080, 0.0185)
η			0.1016	0.0973	0.1066		(0.0042, 0.3613)	
ξ			3.1331	1.3937	4.5346		(0.0775, 12.337)	

MCMC convergence diagnostics were performed through graphical analysis. The kernel density estimates of the posterior distributions demonstrate smooth and well-behaved distributions that confirm thorough parameter space exploration, while the trace plots demonstrate stable mixing behavior without noticeable trends, confirming proper mixing and convergence. These diagnostic plots are presented are presented in S3–S4 Figs.

**Fig 11:**
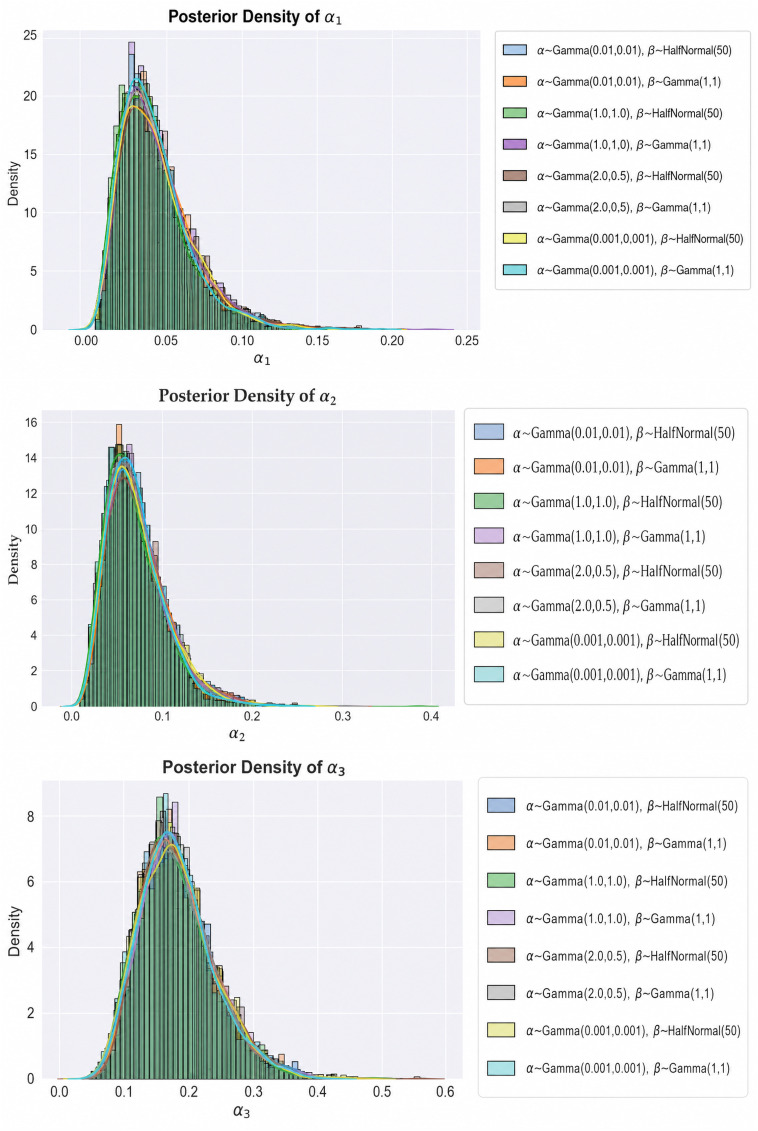
Posterior density for parameters $\alpha_{1},\alpha_{2}$, and $\alpha_{3}$ under different alternative priors (Breakdown data).

**Fig 12:**
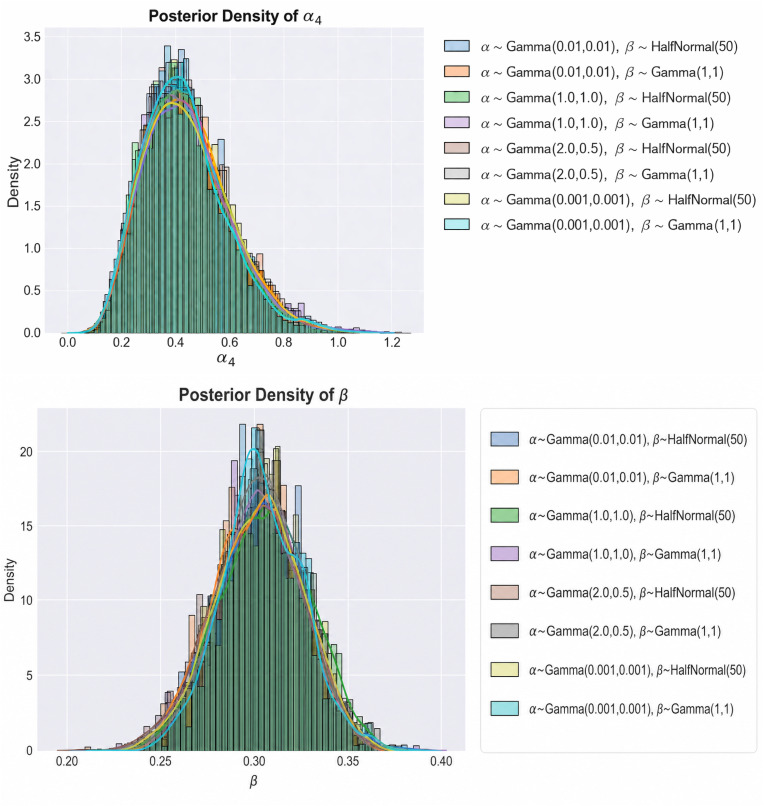
Posterior density for parameters $\alpha_{4}$ and β under different alternative priors (Breakdown data).

**Fig 13:**
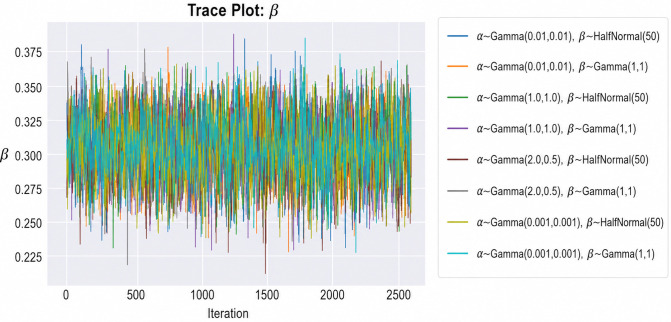
Trace plot for β parameter under different prior alternatives (break down data).

The robustness of the Bayes inference is demonstrated in Figs 11 and 12. Even with extremely diffuse priors such as Gamma(0.001,0.001), the posterior density is almost similar to that obtained under more informative priors. From Table 17, across all priors, β posteriors nearly coincide (CV ≈ 0.004; relative range ≈ 0.014), indicating β is effectively insensitive to prior specification. The $\alpha_{i}$ posteriors for breakdown datasets show low sensitivity (CV typically 0.008–0.020; relative range < 0.07), with tight overlap of density curves. Hyperparameters η and ζ exhibit only modest variation (CV ≈ 0.040, and 0.009, respectively); the posterior mass shifts are small and do not affect the main inferences. β trace plot in Fig 13 demonstrates stable mixing and convergence to the same region across all prior choices, underscoring the model’s robustness. These results ensure that the posterior estimates for β and $\alpha_{i}$ are controlled primarily by the data rather than the priors, confirming that this study’s inferential conclusions remain stable under substantial prior perturbations.

**Table 17:** Breakdown sensitivity metrics for model parameters for breakdown data.

Parameter	CV of Means	Relative Range
β	0.004280	0.014290
$\alpha_{1}$	0.021306	0.066143
$\alpha_{2}$	0.020067	0.067387
$\alpha_{3}$	0.008594	0.027027
$\alpha_{4}$	0.008409	0.025200
η	0.040593	0.114332
ξ	0.009359	0.033117

## 8. Conclusion

In this study, we investigated a flexible statistical framework for lifetime data analysis under the BAPCS, assuming the Chen distribution as the underlying model. The model accommodates heterogeneity across experimental or production settings by incorporating a common shape parameter and allowing the scale parameters to vary across groups, thereby capturing DDF. To perform statistical inference, we considered three estimation techniques: MLE, MPSE, and hierarchical Bayesian estimation under symmetric and asymmetric loss functions utilizing the MCMC technique.

Extensive simulation studies were conducted to assess estimator performance in terms of average bias, mean squared error, coverage probability, and average interval width. The results showed that MPSE achieved greater accuracy and stability, especially under complex censoring configurations or small sample sizes, followed by Bayesian estimation under LINEX loss function.

The methodology was further validated using two real-life data sets: one involving cancer survival times and the other involving electrical insulation breakdown. These data sets featured heterogeneous groups and varied censoring mechanisms, which makes them suitable for evaluating the BAPCS design. The Chen distribution successfully modeled the underlying monotonic hazard behavior, and the proposed inference methods effectively captured the DDF. Across both applications, the proposed methods produced consistent parameter estimates, and the Bayesian approach offered narrower credible intervals, reinforcing its practical reliability. MCMC diagnostics confirmed the stability of the Bayesian procedure, with smooth unimodal posteriors and well-mixed trace plots indicating proper convergence. Additionally, the profile log-likelihood surfaces were smooth and unimodal, supporting the existence and uniqueness of the MLEs.

Although this study focused on the Chen distribution, the proposed framework can be extended to other lifetime models, including the Kumaraswamy, inverse Weibull, and Burr-XII distributions. Similarly, the results can be generalized to other censoring mechanisms, such as progressive first-failure censoring and generalized hybrid schemes. Future research may also explore competing risks models under BAPCS, as well as accelerated life-test designs. Another promising direction is the study of optimal test plan designs that account for DDF across test units.

## Supporting information

S1 Fig Kernel density for the parameters obtained from the MCMC algorithm for cancer patients data. (PDF)

S2 Fig Trace plots for the parameters obtained from the MCMC algorithm for the cancer patients data. (PDF)

S3 Fig Kernel density for the parameters obtained from the MCMC algorithm for the time-to-breakdown data. (PDF)

S4 Fig Trace plots for the parameters obtained from the MCMC algorithm for the time-to-breakdown data. (PDF)
