# Supplementary material for: Classical and Bayesian reliability inference for the chen distribution under a block-adaptive progressive hybrid censoring plan
Source: PLoS One. 2026 Aug 14;21(8):e0355463. doi: 10.1371/journal.pone.0355463 (PMC13475907; doi:10.1371/journal.pone.0355463)
Supplement: Supplementary file 3 [file pone.0355463.s003.pdf]

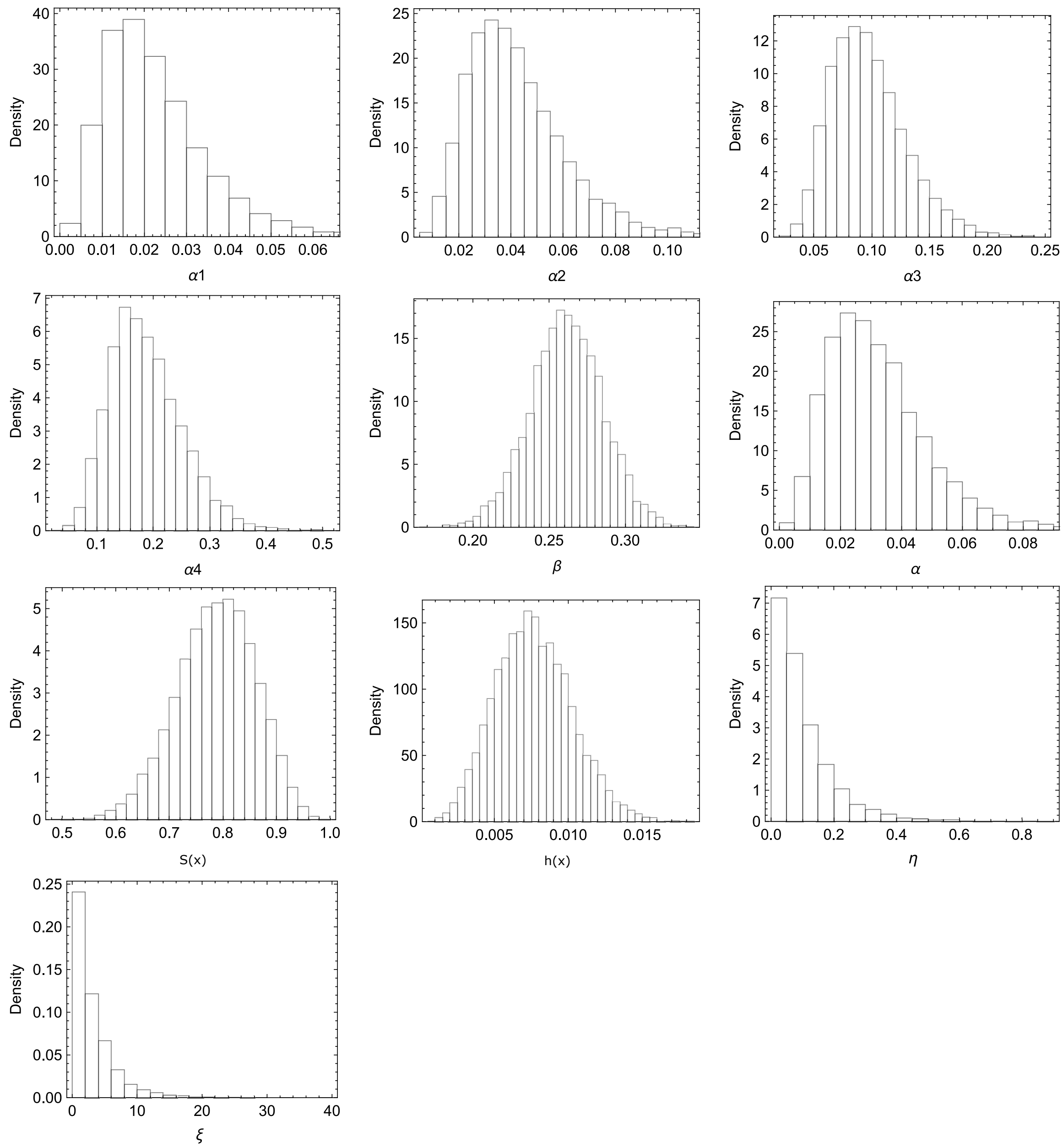

S3 Fig. Kernel density for the parameters obtained from the MCMC algorithm for the time-to-breakdown data.
